# Emergent Synergistic Grasp-Like Behavior in a Visuomotor Joint Action Task: Evidence for Internal Forward Models as Building Blocks of Human Interactions

**DOI:** 10.3389/fnhum.2019.00037

**Published:** 2019-02-06

**Authors:** Lin Lawrence Guo, Namita Patel, Matthias Niemeier

**Affiliations:** ^1^Department of Psychology, University of Toronto Scarborough, Scarborough, ON, Canada; ^2^Centre for Vision Research, York University, Toronto, ON, Canada

**Keywords:** forward model, motor control, grasping, joint actions, interpersonal coordination

## Abstract

Central to the mechanistic understanding of the human mind is to clarify how cognitive functions arise from simpler sensory and motor functions. A longstanding assumption is that forward models used by sensorimotor control to anticipate actions also serve to incorporate other people’s actions and intentions, and give rise to sensorimotor interactions between people, and even abstract forms of interactions. That is, forward models could aid core aspects of human social cognition. To test whether forward models can be used to coordinate interactions, here we measured the movements of pairs of participants in a novel joint action task. For the task they collaborated to lift an object, each of them using fingers of one hand to push against the object from opposite sides, just like a single person would use two hands to grasp the object bimanually. Perturbations of the object were applied randomly as they are known to impact grasp-specific movement components in common grasping tasks. We found that co-actors quickly learned to make grasp-like movements with grasp components that showed coordination on average based on action observation of peak deviation and velocity of their partner’s trajectories. Our data suggest that co-actors adopted pre-existing bimanual grasp programs for their own body to use forward models of their partner’s effectors. This is consistent with the long-held assumption that human higher-order cognitive functions may take advantage of sensorimotor forward models to plan social behavior.

**New and Noteworthy:** Taking an approach of sensorimotor neuroscience, our work provides evidence for a long-held belief that the coordination of physical as well as abstract interactions between people originates from certain sensorimotor control processes that form mental representations of people’s bodies and actions, called forward models. With a new joint action paradigm and several new analysis approaches we show that, indeed, people coordinate each other’s interactions based on forward models and mutual action observation.

## Introduction

**FIGURE 1:**
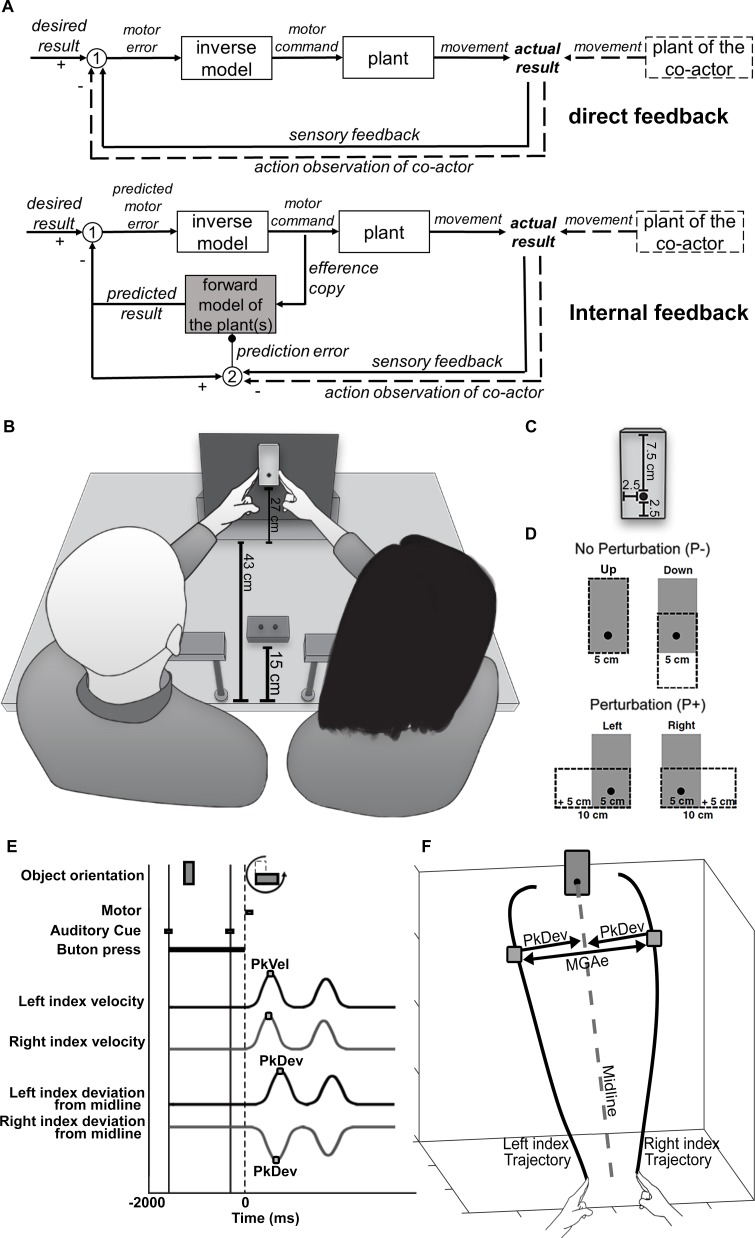
**(A)** Flow charts of sensorimotor control systems. *Top chart*: direct feedback. Sensory feedback conveying information about the current state of the body/the actual result is compared with a desired result (white circle 1). The resultant difference (“motor error”) is converted by a motor control component, called an “inverse model,” into a motor command to be sent to a plant. The plant could be a single muscle or a larger part of the body. It generates a movement that alters the actual result until the difference between sensory feedback and desired result is reduced to an error of zero. *Bottom chart*: internal feedback. Here the (predicted) motor error is computed from internal information rather than sensory information, which is gathered from a copy of the motor command. This “efference copy” is sent to a virtual representation of the plant (“forward model”) to calculate a virtual sensory signal that predicts the effect of the physical plant on the actual result (“predicted result”). In addition, the predicted result is compared with sensory feedback from the actual result (white circle 2) to generate a teaching signal (“prediction error”) that trains the forward model, so to improve its predictions in the future (small black circle). Direct and internal feedback systems could both incorporate the movements of another person (“plant of the co-actor”), e.g., during joint actions, through action observations, a special kind of sensory feedback. Note that action observation would have a transient, real-time (“online”) influence in the direct feedback system whereas it would have a longer lasting, offline influence in the internal feedback system. As a teaching signal it would serve to shape the forward model into an offline representation of both co-actors’ plants. **(B)** Overview of the experimental setup. **(C)** The target object in its vertical (“upright”) orientation. The black dot marks the center of rotation and the point of fixation. **(D)** Object rotation/perturbation levels. When the object rotates from upright to left or right, the width (task-relevant dimension) increases by 5 cm. **(E)** Timeline of a trial. PkVel: Peak finger velocity; PkDev: Peak finger deviation from midline. **(F)** Calculation of MGAe; MGAe was determined trial by trial as the distance between the two index fingers at peak deviation from midline (PkDev). The midline was a path from the midpoint between the two index fingers at the start position to the object’s center of rotation. The midline was calculated for each pair of participants separately.

When we join a partner to lift a table, tango over a dance floor, or when we play the tuba in a musical band, we need to align our own actions with those of others. The coordination of these interactions builds on sensorimotor coordination mechanisms for our own body’s more than 600 muscles. To control our muscles, our brain uses feedback systems in which muscle activity occurs until a desired state of the body or of a body part has been attained, for example until a lower arm is bent as much as required to lift an object (e.g., Todorov and Jordan, 2002; Scott, 2004; Franklin and Wolpert, 2011). To this end, feedback systems need to estimate the actual state of the body in one of two possible ways (Figure 1A). One is called “direct feedback” because it uses online sensory information from our muscles and skin, eyes and ears. However, senses can only signal body states that have already occurred, and often that is too slow for fast action control (Hermosillo et al., 2011; Frost and Niemeier, 2015). Alternatively, “internal feedback” predicts future body states before any movement has occurred. It uses cues such as efference copies of the central nervous system’s own motor commands (Sommer and Wurtz, 2008) and prior experience in internal, virtual representations of the body to anticipate the effects of impending motor activity. Crucially, it has been argued that these internal representations, also called forward models, have developed the ability to incorporate other people’s bodies and minds. They might coordinate physical interactions with other individuals as well as abstract interactions and, thus, constitute some of the seed mechanisms of human social cognition (Wolpert et al., 2003).

A possible connection between forward models and planning processes in social contexts is appealing and would offer fundamental insights into the origins of high-level cognitive functions of the human mind. However, it would be insufficient to take the similarities between forward models and social cognition at face value. Also, as we will argue, as of yet there is limited empirical support for this idea. More evidence is needed to decide whether social cognitive functions are indeed based on sensorimotor forward models.

To test whether it is feasible that social behavior is governed by forward models, here we inspected a relatively simple form of behavior that lends itself more readily to the scrutiny of sensorimotor methods. We tested pairs or dyads of human participants during specific forms of physical interactions, commonly referred to as “joint actions.” Joint actions are defined as behaviors that coordinate one’s own actions with those of others in time and space while pursuing a common goal with the potential to dramatically increase the scope of the outcome (e.g., Clark, 1996; Sebanz and Knoblich, 2009; Sacheli et al., 2013).

Proficient coordination of joint actions likely hinges on action observation and action understanding (Johansson, 1973; Fadiga et al., 1995; Frith and Frith, 1999). It is generally held that these processes involve the fronto-parietal mirror neuron networks (e.g., Fadiga et al., 1995, 2002; Gallese et al., 1996; Rizzolatti and Arbib, 1998; Iacoboni et al., 1999, 2005; Grèzes et al., 2001; Gallese, 2003; but see, e.g., Turella et al., 2009), and it has been argued that mirror neuron networks could function as forward models and/or inverse models (see Figure 1A; e.g., Miall, 2003), which both might closely interact with one another (Wolpert and Kawato, 1998). The close connection between action understanding and motor systems is further supported by the observation that action acquisition appears to precede action prediction and understanding in infants (e.g., Falck-Ytter et al., 2006; Kanakogi and Itakura, 2011), that adults seem to use their own action plans to understand other people’s actions (Flanagan and Johansson, 2003), and that magnetic stimulation of motor cortex will impact action understanding (Elsner et al., 2013). In addition, humanoid movements might be critical for action understanding (Sciutti et al., 2014).

Given the work on action understanding, it seems fitting that people can extract the goal of an observed movement early on (Ansuini et al., 2016; Vaziri-Pashkam et al., 2017). Further, they are capable of monitoring each other’s actions to a level that approaches the observation of one’s own actions (Loehr et al., 2013; also see Jokisch et al., 2006), so much so that people seem to inadvertently create shared task representations (Flanagan and Johansson, 2003), even when these representations are detrimental to their own performance (Schmidt et al., 1990; Kilner et al., 2003; Sebanz et al., 2003, 2005) or result in movements in violation of sensorimotor laws (Fine and Amazeen, 2011) although such effects might not be (entirely) automatic because action observations vary as a function of joint attention (Shockley et al., 2003).

To facilitate mutual action observation and coordination, people may intentionally employ various strategies. Based on strategies they may act in anticipation of their partner’s task (Knoblich and Jordan, 2003; Becchio et al., 2008a; Ray and Welsh, 2011; Vesper et al., 2013) or reduce behavioral variability, possibly to make their actions more predictable for their partner, especially if they assume a leadership role within the dyad (Sacheli et al., 2013). Further, it has been suggested that people may establish special codes or signals (Badino et al., 2014; Candidi et al., 2015) and sensory channels of communication (Reed et al., 2006; Van der Wel et al., 2011). This body of data appears to indicate that people develop coordination strategies with the help of mental simulations of each other’s actions in support of the idea that forward models facilitate interactions. Alternatively however, the interaction strategies could have been developed entirely based on cognitive mechanisms, given that all paradigms reporting coordination strategies make action coordination available to conscious cognitive control.

To elaborate: cognitive control is well known to aid motor learning (e.g., Sherwood and Lee, 2003). With its access to a wealth of cognitive functions, including various forms of mental imagery, it endows the human brain with an extraordinary capability to generate functional behavior in a multitude of novel situations. Thus, because action control can be enabled by cognitive functions (or even attitudes, e.g., Becchio et al., 2008b), it becomes difficult to disentangle causalities: perhaps cognitive functions give rise to forward models instead of forward models enabling sensorimotor interactions and social cognition. To avoid such a “chicken-and-egg problem” and to demonstrate that forward models do give rise to interactions it is for methodological reasons important to examine interactions where coordination through conscious cognitive control can be ruled out. For example, task instructions could direct people’s attention away from certain features of the joint actions so that their coordination is less likely governed by conscious control.

Also little conscious control is involved where people coordinate their joint actions during the same trial, in immediate response to their partner’s movements when the responses are too fast for conscious control (e.g., Solnik et al., 2016). Such “same-trial coordination” occurs, for example, in scenarios where co-actors are mechanically coupled (Harrison and Richardson, 2009) so that partners can rely on fast somatosensory channels and perhaps even spinal reflexes. Even without spinal reflexes, vision-based same-trial coordination is possible. For example, there is good evidence for action interference or mimicking where co-actors involuntarily show signs of copying another person’s movements (see, e.g., the “followers” in Sacheli et al., 2013). As another example, Georgiou et al. (2007) found that participants coordinated the timing of their hand movements when they were asked to make joint reach-to-grasp movements such that, from trial to trial, certain features of one person’s movements occurred earlier or later in coordination with their partner’s timing, apparently because the partners were quick enough to adjust their timing based on action observation.

However, any form of such same-trial coordination of joint actions could be attained through online action observation within direct feedback systems (see Figure 1A). Thus, whether internal feedback and forward models play any part in joint actions with same-trial coordination is difficult to tell.

To avoid the difficulty, one can inspect joint action features that cannot be observed in real time so that same-trial coordination becomes impossible. In that case, coordination in the future might still be possible, but only if co-actors form a memory of one another’s movement characteristics. One example for such a memory could be forward models (Figure 1A). Specifically, an internal feedback system involved in coordinating joint actions could use action observations to train its forward model. Thus, the forward model would turn into a memory of past action observations. Over time, the co-actors’ movements would settle in a state where each person’s general trend of moving is coordinated with their partner’s average movements, henceforth called “average-trial coordination.” In sum, without same-trial coordination, direct feedback could be ruled out. Any persisting average-trial coordination could then indicate internal feedback. (Average-trial coordination could also come from conscious cognitive control that would have to be ruled out as well, as mentioned earlier).

The aim of the present study was to test whether it is feasible that internal sensorimotor feedback with its forward models equips the human brain with the mental tools to embody other people’s actions and intentions for sensorimotor interactions and eventually abstract forms of interactions and social cognition (Wolpert et al., 2003). To this end, we investigated whether co-actors can implicitly align their actions based on average-trial coordination. We employed a novel cooperative object lifting task. We asked two partners to move the index fingers of their left or right hand, respectively, to lift an object (Figure 1B) as if a single person reached and grasped the object bimanually with their two index fingers (e.g., Le and Niemeier, 2013a,b; Le et al., 2014). Grasping tasks have been used as paradigms particularly suitable to study goal directed behavior (Hamilton and Grafton, 2006; Sacheli et al., 2015) with well-understood underlying neural mechanisms (Culham et al., 2003; Davare et al., 2006; Grafton, 2010; Monaco et al., 2013). Also, our task included timing features where we could expect that same-trial coordination is possible (as shown for a related task, Georgiou et al., 2007). Crucially, we expected same-trial coordination to be impossible for peak velocity and maximum movement curvature or peak deviation. These two central features of reach-to-grasp movements can be observed only *post hoc*, once the entire movement is largely completed. Further, the task provided a scenario where people had ample sensorimotor experience to act on their own while at the same time they were likely unaware of the movement features of bimanual grasping in detail and certainly had no experience with sharing the task in the form of a joint lifting task with another person. In addition, because we anticipated that co-actors would exhibit coordinated grasp-like behavior, we sought to disrupt grasp-specific coordination using object perturbations as they are commonly used to target the grasp component of reach-to-grasp movements in individual people (Paulignan et al., 1991; Glover et al., 2005; Tunik et al., 2005; Le et al., 2014; Zaal and Bongers, 2014). Finally, data from the cooperative lifting condition were compared (a) to a competition condition where participants raced to reach and touch the sides of the object without lifting it, and (b) to a separate control experiment where individual participants performed bimanual grasps on the object. We expected that dyads should exhibit joint lifting behavior that is largely akin to bimanual grasping of an individual person. Specifically however, we expected dyads should show no same-trial coordination of peak velocities and peak deviations. Instead, these two movement features could show average-trial coordination, indicative of forward models. Alternatively, the features should be entirely uncoordinated if joint actions could not rely on forward models.

## Materials and Methods

### Participants

Twenty-two healthy undergraduate students from the University of Toronto at Scarborough gave their written and informed consent to participate in the joint lifting experiment (17 females; median age = 18; *SD* = 0.8) and were pseudorandomly sorted into 11 pairs. An a-priori power analysis using one-sample comparison effect sizes found in measures of grasp proficiency (minimum *d* = 5.858; Le and Niemeier, 2013a,b) revealed that a sample size of 3 would provide sufficient statistical power at the recommended 0.80 level (G^∗^Power; Cohen, 1988; Faul et al., 2007). Given that coordinating a grasp-like movement across individuals could induce additional variability, we decided on a minimum sample size of 10. Comparable sample sizes are typically found in grasping studies with TMS (e.g., Tunik et al., 2005; Le et al., 2014, 2016). In addition, for a control experiment another 11 undergraduate students from the University of Toronto at Scarborough participated after giving their written and informed consent (6 females; median age = 19; *SD* = 0.9). Participants in both experiments were recruited at about the same time (e.g., whenever one of the two participants of the dyadic experiment failed to show up, the other person was tested in the bimanual experiment). Participants in both experiments were right handed (Oldfield, 1971) and had normal or corrected to normal vision. All procedures were approved by the Human Participants Review Sub-Committee of the University of Toronto and conformed to the ethical standards laid down in the Declaration of Helsinki.

### Procedure and Apparatus

Dyads performed two tasks. In one task, they worked together to lift an object by reaching to, and then pushing against the lateral sides of the object so as to remove it from its mount (JOINT task; see Figure 1). In the other task, they were asked to reach and touch the sides of the object while trying to be faster than their respective partner (Competition task; COMP). For both JOINT and COMP task, the participant on the left used his or her left index finger and, as a support, the left middle finger, and the participant on the right used his or her right index and middle fingers. In pilot tests we found that participants quickly learned to perform the task without dropping the object. Therefore, we had participants perform 32 practice trials prior to each condition, and then one block of 80 trials for each of the two tasks. The order of blocks was randomly counterbalanced across pairs (order did not produce significant differences).

During the experiment, the pair sat side by side and rested their heads on adjustable chin-rests (average height from table’s surface = 29.4 cm; *SD* = 3.1 cm), separating the heads by 40 cm (Figure 1B). The set-up allowed participants to view, in their visual periphery, their partner’s hand movements. For hand movement tracking, three infrared motion capture cameras (Qualisys, sampling rate: 240 Hz) recorded three-dimensional movement trajectories from spherical passive infrared markers (10 mm across) taped to the index fingers and wrists of the participants’ performing hands. Eye movements were not recorded, but participants were asked to fixate on a fixation dot on the target object (fixation was implicitly encouraged because the object rotated around the fixation point while participants moved their hands to it, see below). As target object, we used a gray rectangular block (front side: 5 cm × 10 cm; thickness: 5 cm) that was mounted on the rotating shaft of a motor (SureStep STP-MTRH-23079, Automation Direct, Atlanta, GA, United States; see Figure 1B) such that it was located 43 cm in front of, and along the midline between the two participants, and 27 cm above the table’s surface. The front side of the object tilted backward, making it roughly orthogonal to the participants’ line of sight. Furthermore, the object’s rotation axis, aligned with the fixation dot, was chosen slightly off center, 2.5 cm away from three of the sides of the object (Figure 1C).

In both JOINT and COMP conditions, trials began with a low-pitched beep that instructed co-actors to use the index finger of his or her performing hand to press and hold one of two buttons on a box that was placed 15 cm in front of, and along the midline between the two participants (distance between buttons: 3 cm). This triggered the motor to rotate the target object to its vertical start orientation, that is, with the front narrow edge facing the ground and with the object’s longer end upward. Five-hundred to 1500 ms later, a high-pitched beep cued the participants to initiate movement (Go signal, Figure 1E). In trials with perturbations of task-relevant object-size (P++), release of start buttons (movement onset) triggered the motor to quickly rotate the object by 90° or 270° clockwise (cw) or counterclockwise (ccw) around the object’s rotation axis. Perturbation occurred in 50% of the trials. In trials without perturbations (P-) the object rotated by 180° or 360° cw or ccw. This way, all P+ and half of all P- trials rotated the object on average by the same amount but P+ trials increased the object’s task-relevant horizontal width by 5 cm on either the left side (90° ccw or 270° cw) or the right side (90° cw or 270° ccw), thus requiring the respective participant to adjust hand trajectories laterally. In contrast, P- trials kept the object’s horizontal width the same (360° rotations turned the object back to its “upright” start orientation, 180° rotations turned the object “downward”; see Figure 1D) so that none of the two participants had to adjust hand trajectories laterally. Perturbation paradigms often change object size (along either the vertical or horizontal axis) from small to large (Tunik et al., 2005; Le et al., 2014; Zaal and Bongers, 2014). Perturbations in the opposite direction have been found to change the timing of movement adjustments (e.g., Glover et al., 2005).

#### Control Experiment

To examine similarities between dyads performing joint lifting and individuals performing bimanual grasps, we conducted a control experiment where individual participants grasped and lifted the object with the index fingers of both hands (and with their middle fingers as an additional support). The experimental protocol was identical to the JOINT condition. Data from bimanual grasping were analyzed the same way as joint lifting (see section “Data Analysis”).

### Data Analysis

Recordings of index finger positions were preprocessed in the Qualysis software and further analyzed with custom-written MATLAB (The Mathworks, Natick, MA, United States) scripts. The data were visually inspected to identify and exclude invalid trials for incompleteness or noisy hand trajectories due to artifacts (JOINT: 6.7%; COMP: 7.1%).

For valid trials, we determined the onset of movements as the point at which index finger velocity exceeded 5% of the respective peak velocity (PkVel, Figure 1E). The time between movement onset and Go signal reflected reaction times (RTs). Note, however, that movement onset preceded one of our experimental manipulations, the object rotations/perturbations (in fact, the button release triggered the object rotations). Therefore, we only briefly inspected RTs (average RT_JOINT_ = 311 ms; average RT_COMP_ = 291 ms; RTs submitted to a repeated-measures ANOVA with factors Task [JOINT vs. COMP] and Hand [left vs. right] produced no significant results: *F*’s ≤ 2.44, *p* ≥ 0.149) and excluded RTs from any further analysis.

To characterize the kinematic profiles of the JOINT and COMP movements we treated them as if they were grasp movements. That is, we focused on two kinematic measures that were calculated for each trial and that are known to represent reach-to-grasp movements well (e.g., Jeannerod, 1984; for that reason called “reach-to-grasp kinematics” henceforth). The first measure, PkVel, is a kinematic landmark for reach movements (Jeannerod, 1984; Gentilucci et al., 1991). Our second measure, peak deviation from the midline (PkDev), captured the “grasp-like” component of the movement. PkDev was defined as the largest perpendicular distance between the index finger and a path between the two participants, extending from the midpoint between the two index fingers at their starting positions to the object’s center of rotation (Figure 1E,F). Further, we defined a “maximum grip aperture equivalent” (MGAe) as the shortest spatial distance between the coordinates of the co-actors’ index fingers at their respective PkDev (see Figure 1F). A more common measure would be “maximum grip aperture” (MGA), i.e., the largest distance between the index and the thumb as they approach an object (Jakobson and Goodale, 1991; Smeets and Brenner, 1999; Le and Niemeier, 2014), or between the index fingers in bimanual grasps (Le and Niemeier, 2013a,b; Le et al., 2014, 2016). However, MGA confounds spatial distance between fingers with their temporal misalignment which could be significant because two co-actors are likely less well coordinated than a single person so that MGA would turn into a measure of poor timing that inflates correlations between spatial and temporal measures. In contrast, PkDev and MGAe are purely spatial measures (nevertheless, the correlation between MGA and MGAe was high, *r* = 0.94). Further, to assess co-actors’ coordination in time, we examined the time at which co-actors reached their PkDev and PkVel in each trial as measures of temporal coordination (e.g., Georgiou et al., 2007). All variables showed non-redundant properties (see differential object perturbation effects and factor analyses in the Results).

We looked at the scaling of MGAe relative to object sizes (i.e., gain = [MGAe of P+ trials – MGAe of P- trials] / [object width in P+ trials – object width in P- trials]; Le and Niemeier, 2014; for a review, see Smeets and Brenner, 1999), with gains around 0 indicating poor grasp performance, and gains closer to 1 reflecting more functional grasps.

Furthermore, we tested whether co-actors made similar movements in that the two partners might show similar movement features (i.e., similar PkDev, PkVel, tPkDev, and/or tPkVel values) as signs of same-trial or average-trial coordination (see section “Introduction”). Same-trial coordination means that co-actors adjust a feature of their own movements to that feature of their partner within the same trial. Consequently, from trial to trial, the two co-actors’ should exhibit movement features that covary. In other words, we should observe correlations within each dyad. We calculated within-dyad correlations for the kinematic and timing variables; for all rotation conditions (up/down/left/right) and the task conditions (JOINT/COMP) separately. Next, we converted the coefficients to Fisher’s *z*-values and averaged across P+ (left/right) and P- conditions (up/down). These data were submitted to repeated-measures ANOVAs with factors Perturbation and Task. Furthermore, *t*-tests were used to probe for significance relative to zero.

Also, we tested whether the kinematic and timing variables showed signs of average-trial coordination. That is, over time, co-actors might be able to learn one another’s movement features and, thus, settle in a state where each person’s general trend of moving is coordinated with their partner’s average movements. To look at average-trial coordination we calculated cross-dyad correlations. Specifically, for each co-actor separately we averaged PkDev, PkVel, tPkDev, and tPkVel variables over trials for each rotation (up/down/left/right) and task (JOINT/COMP) condition separately, and then correlated the results across dyads for each condition separately (correlations for up and down rotations as well as for left and right rotations were then averaged to form P- and P+ conditions). For correlation data, no parametric inference test exists. Instead, we used bootstrapping to randomly resample, with replacement, the 11 sets of dyads and then recalculate Fisher’s *z*-transformed correlations or correlation differences. We repeated these steps 10,000 times to compute confidence intervals and to decide whether a null hypothesis could be rejected with an alpha error of 5%. Furthermore, we used factor analyses and cross-correlations *post hoc* to examine the mutual influence of the different variables in the different experimental conditions.

For the bimanual control experiment we submitted kinematic and timing variables to equivalent analyses to look for functional grasps as well as within-subject and cross-subject correlations. We expected all correlations to be significant, including within-subject correlations for the kinematic variables because unlike dyadic coordination, bimanual coordination within a person can rely on internal signals from efference copies.

## Results

### Control Experiment: Bimanual Grasping

**FIGURE 2:**
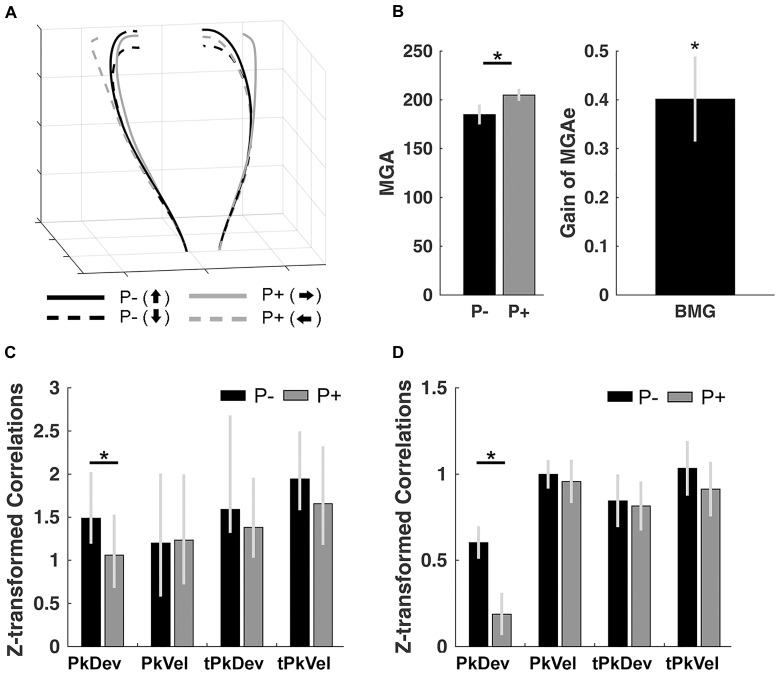
Control experiment results. **(A)** Normalized trajectories of bimanual grasping for the left and right hands from movement onset to object contact. P–, no perturbation; P+, perturbation. Arrows pointing up/down/left/right: final orientation of the object. **(B)** Size of MGAe during perturbation (P+) and no-perturbation (P–) trials, and the scaling of MGAe to different object widths. Gain = [MGA of object width in P+ trials – MGA of object width in P– trials] / [P+ object width – P– object width]. Error bars indicate standard error. **(C)** Synchrony of peak finger deviation (PkDev), peak finger velocity (PkVel), timing of peak finger deviation (tPkDev), and timing of peak finger velocity (tPkVel) between the left and right hands in each of the perturbation conditions (P–, no perturbation; P+, with perturbation) quantified using correlations across persons (Fisher’s *z*-transformed). Error bars indicate 95% bootstrapped confidence interval. **(D)** Same-trial coordination quantified using within-subject correlations (Fisher’s *z*-transformed) of reach-to-grasp kinematics and timing variables in each of the perturbation conditions. ^∗^ Indicates significance at *p* < 0.05.

For comparison purposes we first report the results from the bimanual reach-to-grasp task performed by a single person to then present dyadic movements in the subsequent Results sections. During bimanual grasping participants moved their hands on curved trajectories (Figure 2A for average trajectories aligned in percent of total travel time; subsequent analyses extracted data from individual trials) as commonly observed (Le and Niemeier, 2013a,b) (Supplementary Figure S1 for a quantification of the kinematics and timing variables of the control experiment). Also as expected, bimanual grasping showed features indicative of functional grasps: MGAe values were smaller without perturbations (up and down rotations averaged) than with perturbations [left and right rotation conditions averaged; *t*(10) = 4.61, *p* < 0.001, *d* = 7.226, Figure 2B], and increased with the horizontal object size with a gain of 0.401, which is significantly larger than zero [*t*(10) = 4.61, *p* < 0.001,

*d* = 7.226]. Note that the gain of MGAe observed here is smaller than the values observed in previous bimanual grasping studies (Le and Niemeier, 2013a,b), likely due to the dynamic nature of the perturbation paradigm.

Next, to inspect same-trial and average-trial coordination of the left and right hand, we calculated within-subject and cross-subject correlations (see section “Materials and Methods”), respectively. Across participants, left and right hand movements were significantly correlated for all kinematics and timing variables (for bootstrapped 2.5 to 97.5% confidence intervals see Figure 2C; the interval closest to 0: [1.0451, 1.778]; see section “Materials and Methods” for details on bootstrapping). Furthermore, we observed a reduction in the correlation of PkDev in the perturbed condition (i.e., the correlations for the left and right rotation conditions averaged; confidence interval of bootstrapped correlation difference: [0.1832, 0.7451]) but not for any other variable (Supplementary Figure S2 plots the correlations within gliding windows over trials).

Next, we computed within-subject correlations of the kinematics and timing variables. Correlations for all conditions were significantly greater than 0 (*t*’s > 5.190; *p*’s < 0.001 after serial Bonferroni correction; Figure 2D). Further, we observed a perturbation effect for PkDev; left and right hand movements were less similar in trials where objects perturbed to the left or right sides [*t*(10) = 2.649; *p* = 0.024; *d* = 0.780].

In sum, bimanual grasp movements showed well-known functional MGAe gains. Signs of same-trial and average-trial coordination were observed for all kinematic and timing variables, as expected. Also, we found specific perturbation effects on the coordination of the PkDev data.

### Dyadic Movements: Basic Analysis

**FIGURE 3:**
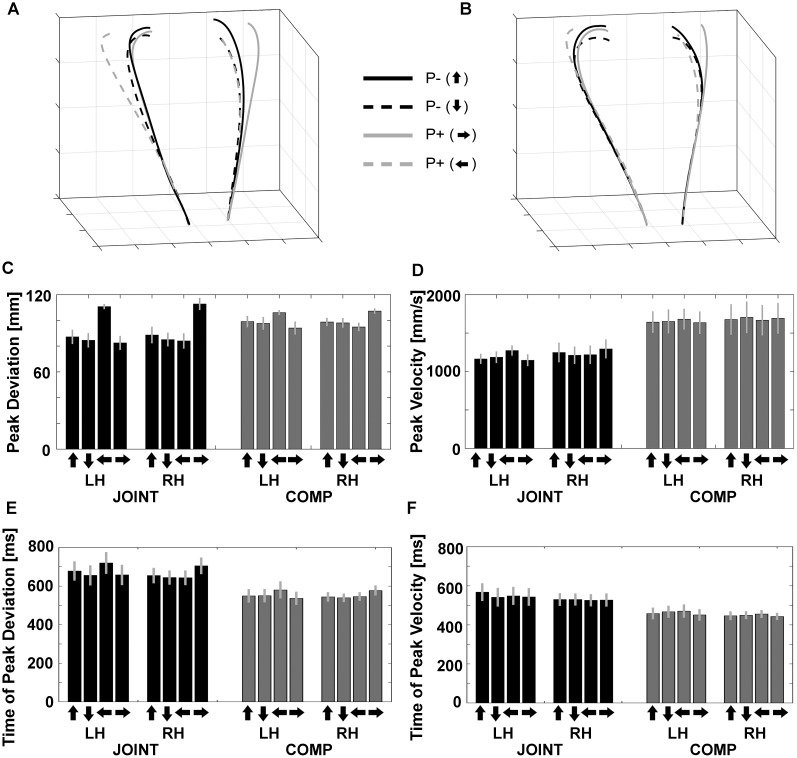
Normalized trajectories of **(A)** cooperation (JOINT) and **(B)** competition (COMP) for the left and right hands from movement onset to object contact. P–, no perturbation; P+, perturbation. Arrows pointing up/down/left/right denote object rotations based on the final orientation of the object. **(C)** Peak deviations of the left (LH) and right (RH) hands during JOINT and COMP. **(D)** Peak velocities of the left and right hands during JOINT and COMP. **(E)** Peak deviation times of the left and right hands during JOINT and COMP. **(F)** Peak velocity times of the left and right hands during JOINT and COMP. Arrows denote end positions of objects after the respective up-/down-/left-/rightward rotation.

Dyadic movements, especially during the JOINT task were similar to bimanual grasping in several important ways: Co-actors moved their hands on similarly curved trajectories during the JOINT and the COMP task with peak deviations during the second half of the trajectory as observed during bimanual grasping (Figure 3A,B for average trajectories aligned in percent of total travel time).

“Reach-to-grasp” kinematics and timing variables were each evaluated with a repeated-measures ANOVA with factors Task (JOINT vs. COMP), Hand (left vs. right), and Object rotation (up/down/left/right). Peak deviation (PkDev; Figure 3C) exhibited an influence of Object rotation [*F*(1.42,14.23) = 14.95, *p* = 0.001 after Greenhouse Geisser correction, GGC, *$\eta_{p}^{2}$* = 0.599] and of the Rotation-by-Task interaction [*F*(1.92,19.18) = 17.09, *p* < 0.001 after GGC, *$\eta_{p}^{2}$* = 0.631], in that horizontal object rotation conditions increased PkDev more than vertical rotation conditions, especially during the JOINT task. Furthermore, the Rotation-by-Hand interaction [*F*(1.26,12.59) = 44.50, *p* < 0.001 after GGC, *$\eta_{p}^{2}$* = 0.817] as well as the 3-way interaction [*F*(2.28,22.76) = 26.89, *p* < 0.001 after GGC, *$\eta_{p}^{2}$* = 0.729] reflected complementary effects of left object rotations on the left hand and of right rotations on the right hand, respectively, especially during the JOINT task. This result is consistent with what is observed when a single person uses both hands in this task (Supplementary Figure S1A; see Zaal and Bongers, 2014, for symmetric effects under specific conditions).

Peak velocities (PkVel) were also modulated (Figure 3D): Co-actors moved more quickly during competition than during cooperation [*F*(1,10) = 23.22, *p* = 0.001, *$\eta_{p}^{2}$* = 0.699] as they attempted to be faster than their co-actor. Object rotation had no main effect [*F*(2.78,27.90) = 1.50, *p* = 0.238], but interacted with factor Hand [*F*(1.95,19.50) = 13.98, *p* < 0.001 after GGC, *$\eta_{p}^{2}$* = 0.583], as well as with factors Hand and Task together [*F*(2.26,22.60) = 4.93, *p* = 0.014 after GGC, *$\eta_{p}^{2}$* = 0.330]. This was mostly due to a complementary impact of left and right object rotations on the left and right hand, respectively, and especially during the JOINT task [all other effects: *F*’s < 1.50, *p*’s ≥ 0.238, including the main effect of Hand: *F*(1,10) = 0.14, *p* = 0.715].

Both timing variables yielded smaller values during competition than during cooperation as expected [time of peak deviation, tPkDev: *F*(1,10) = 36.233, *p* < 0.001, *$\eta_{p}^{2}$* = 0.784, Figure 3E; time of peak velocities, tPkVel: *F*(1,10) = 24.406, *p* = 0.001, *$\eta_{p}^{2}$* = 0.709, Figure 3F]. No other effects were found for tPkVel. Object rotation had an effect on tPkDev [*F*(3,30) = 7.062, *p* < 0.001, *$\eta_{p}^{2}$* = 0.414], and interacted with factor Hand [*F*(3,30) = 24.743, *p* < 0.001, *$\eta_{p}^{2}$* = 0.712], as well as with factors Hand and Task together [*F*(3,26.8) = 3.528, *p* = 0.032, *$\eta_{p}^{2}$* = 0.261]. This was mostly due to a complementary impact of left and right object rotations on the left and right hand, respectively, and especially during the JOINT task.

### Dyadic Movements: Grasp-Like Movements During JOINT vs. COMP Trials

**FIGURE 4:**
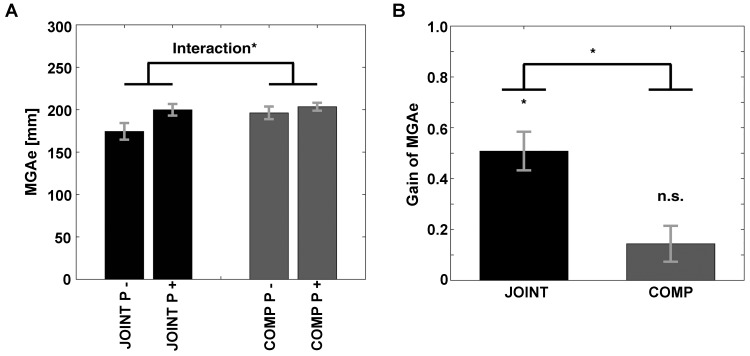
**(A)** Size of MGAe during cooperation (JOINT) and competition (COMP) for perturbation (P+, i.e., left and right object rotations collapsed) and no-perturbation (P–, i.e., up and down rotations collapsed) trials. **(B)** Scaling of MGAe to different object widths for cooperation (JOINT) and competition (COMP). Gain = [MGA of object width in P+ trials – MGA of object width in P– trials] / [P+ object width – P– object width]. Error bars indicate standard error. ^∗^ Indicates significance at *p* < 0.05.

As expected, we found evidence that participants exhibited grasp-like behavior during the JOINT task. That is, the equivalent of MGAe (see Figure 1F) was scaled to the “grasp”-relevant dimension of the object specifically during the JOINT task: First, MGAe values submitted to a repeated-measures ANOVA with factors Task (JOINT vs. COMP) and Perturbation (vertical rotations = P- vs. horizontal rotations = P+) yielded a main effect of Perturbation [*F*(1,10) = 27.17, *p* < 0.001, *$\eta_{p}^{2}$* = 0.731] and a Task-by-Perturbation interaction [*F*(1,10) = 22.73, *p* = 0.001, *$\eta_{p}^{2}$* = 0.695; Task effect: *F*(1,10) = 4.21, *p* = 0.066, *$\eta_{p}^{2}$* = 0.298, Figure 4A]. Thus, MGAe values were smaller without perturbation, and especially so in the JOINT condition. Second, during the JOINT task, MGAe changed with object size with a gain of 0.51, which is significantly larger than zero [*t*(10) = 6.70, *p* < 0.001, *d* = 2.018, Figure 4B], and similar to grasps with the non-dominant hand (Le and Niemeier, 2014), despite the fact that gain here was determined in a perturbation paradigm as opposed to more typical static conditions (also see a similarly reduced value in the control experiment).

The COMP task gain was significantly smaller than the JOINT task gain [*t*(10) = 4.77, *p* < 0.001, *d* = 1.438], and not different from zero [gain = 0.14; *t*(10) = 2.04, *p* = 0.069, *d* = 0.614]. Crucially, the insignificant COMP gain was not due to a speed-accuracy trade-off: rather than negative correlations between gains and average PkVel’s, we found numerical trends in the opposite direction (JOINT: *r* = 0.429; COMP: *r* = 0.777, not significant according to bootstrapping).

### Dyadic Movements: Cross-Dyad Correlations of “Reach-to-Grasp” Kinematics and Timing

Because curvatures of JOINT actions resembled bimanual grasps of an individual person as anticipated, we expected that co-actors should show some of the movement coordination that individual participants had exhibited (Figure 2C,D).

**FIGURE 5:**
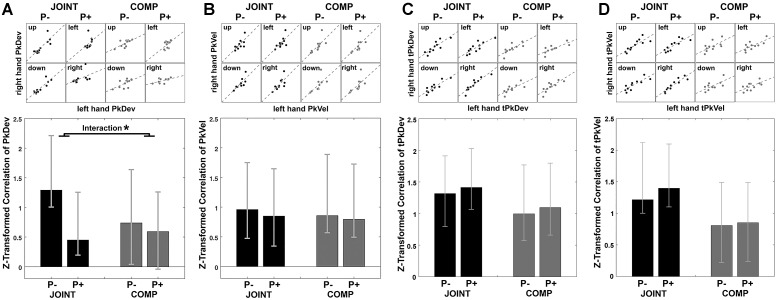
Synchrony of peak finger deviation **(A)**, peak finger velocity **(B)**, timing of peak finger deviation **(C)**, and timing of peak finger velocity **(D)** between co-actors illustrated by scatterplots for cooperation (JOINT) and competition (COMP) in each rotation condition. Each dot in the scatterplots is the averaged variable of each co-actor. Bar graphs display the Task by Perturbation interaction of *z*-transformed correlations for each of the four variables, calculated by [(JOINT P– to JOINT P+) – (COMP P– to COMP P+)]. Error bars indicate 95% bootstrapped confidence interval. ^∗^ Indicates significance as per bootstrapping analysis.

First, we examined cross-dyad correlations as indicators of average-trial coordination (see section “Materials and Methods”). Scatter plots in Figure 5A display each co-actor’s contribution to the MGAe, the PkDev, and the bar graph in Figure 5A presents Fisher’s *z*-transformed correlations of PkDev data in each condition together with bootstrapped confidence intervals (2.5 to 97.5%). Indeed, each co-actor’s PkDev was similar to the PkDev of his or her partner during each condition, and except for COMP P+, all conditions produced significant and positive cross-dyad correlations indicating coordination.

What is more, PkDev correlations were more prominent during the JOINT task, especially during unperturbed trials as the standard condition (also see section “Discussion”). To show this we examined the influence of factors Task (JOINT vs. COMP) and Perturbation (P- vs. P+) on the correlations. In lieu of an ANOVA (which does not apply here), we bootstrapped the differences between correlations. For factor Task [(JOINT P- plus JOINT P+)/2 minus (COMP P- plus COMP P+)/2], the resulting confidence intervals included zero (-0.50, 1.30), i.e., overall JOINT and COMP correlations did not differ significantly from each other. However, a significant factor Perturbation indicated that perturbations in general reduced coordination {[(JOINT P- plus COMP P-)/2 minus (JOINT P+ plus COMP P+)/2]; confidence intervals: [0.17, 0.93]}. Crucially, the Task-by-Perturbation interaction was significant {[(JOINT P- minus JOINT P+) minus (COMP P- minus COMP P+)], confidence interval: [0.12, 1.20]}, thus perturbations were most disruptive in the JOINT condition with JOINT P- yielding the greatest degree of coordination.

PkVel, tPkDev, and tPkVel data displayed correlations that were significantly positive correlations throughout (Figure 5B–D). Indeed, both tasks afforded temporal alignment, either to arrive at similar times at the object or to outmatch the respective other’s efforts to be faster. In addition, factor Task modulated correlations of tPkVel (confidence interval: [0.16, 2.34]), such that tPkVel was more correlated during JOINT than COMP. Additional analyses looking at the change in correlations within time windows gliding across trials only showed small effects: PkDev correlations in the JOINT P- condition slightly increased over the course of the experiment; for the timing variables correlations slightly decreased (Supplementary Figure S3).

In sum, cross-dyad correlations of “reach-to-grasp” kinematics and timing variables showed that the movements of co-actors, averaged over time, were similarly coordinated as those of an individual with a similarly specific effect of perturbation on PkDev values.

### Dyadic Movements: Differences in Same-Trial vs. Average-Trial Coordination Between Timing and “Reach-to-Grasp” Kinematics

**FIGURE 6:**
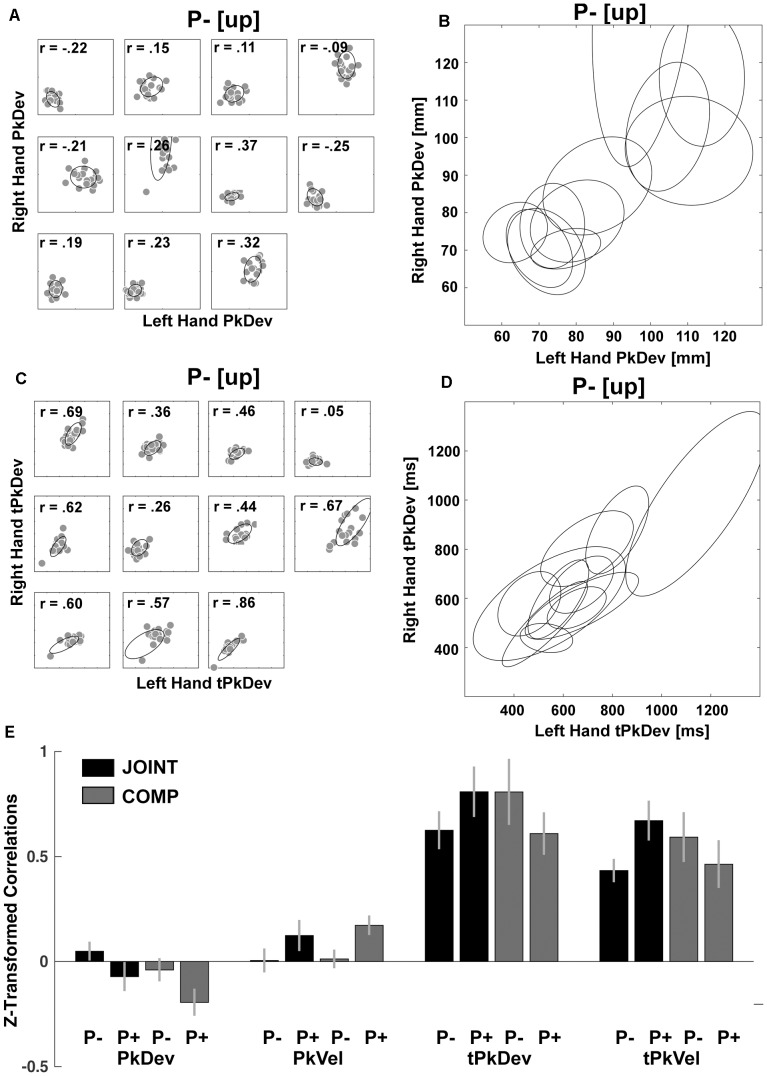
Within-dyads correlations. **(A)** Scatterplots of each dyad’s PkDev in JOINT P– [up rotation condition] with fitted ellipses. **(B)** PkDev ellipses from all dyads. **(C)** Scatterplots of each dyad’s tPkDev in JOINT P– [up rotation condition] with fitted ellipses. **(D)** tPkDev ellipses from all dyads (for ellipse fitting method see Fitzgibbon et al., 1999). **(E)** Fisher’s *z*-transformed correlations of reach-to-grasp kinematics and timing variables in each condition.

However as expected, *within*-dyad correlations revealed that co-actors coordinated “reach-to-grasp” kinematics fundamentally differently from timing variables. For the “reach-to-grasp” kinematics we found within-dyad correlations to be spurious (e.g., Figure 6A compared to Figure 6B). In contrast, timing variables produced sizeable correlations within and across dyads (e.g., Figure 6C,D). We confirmed this with a series of inference tests. First, we converted the correlations with Fisher’s *z*-transformations to make them suitable for parametric testing (Figure 6E). Next the *z*-values entered repeated-measures ANOVAs with factors Perturbation (P- vs. P+) and Task (JOINT vs. COMP) for the “reach-to-grasp” kinematics and timing variables separately. This produced a main effect of Perturbation on PkVel correlations [*F*(1,10) = 7.75, *p* = 0.019; other *F*’s < 1], reflecting a small rise in correlations during perturbations. The Perturbation effect on PkDev correlations was not significant [*F*(1,10) = 4.43, *p* = 0.062; other *F*’s ≤ 2.01, *p*’s ≥ 0.186]. The ANOVA for tPkVel correlations yielded an interaction of Task and Perturbation, with higher correlations in P+ trials than P- trials during JOINT, but lower correlations in P+ trials than P- trials during COMP [*F*(1,10) = 7.071, *p* = 0.024; for tPkDev: *F*(1,10) = 3.801, *p* = 0.080; all other *F*’s ≤ 1.75, *p*’s ≥ 0.215]. Finally, for each variable we averaged the correlations across conditions to test against zero with serial Bonferroni correction. The correlations for the timing variables were significantly larger than zero (*t*’s > 8.710, *p*’s < 0.001), indicating that co-actors coordinated their timing importantly based on same-trial mechanisms (Solnik et al., 2016). PkVel correlations were significant too [*t*(10) = 2.94, *p* = 0.015], but small (equivalent to an *r*-value of 0.08). For PkDev the correlations were significantly smaller than zero [*t*(10) = -2.45, *p* = 0.034]. In addition, to confirm that the within-dyad correlations were not due to task properties (e.g., stimulus-driven behavior that is common to most participants), we randomly mixed the dyads and re-ran the correlation analyses. The resulting correlations were not significantly greater than 0 (*t*’s < 0.376; *p*’s > 0.646). Thus, online mechanisms had little influence on coordination of “reach-to-grasp” kinematics, and the correlation patterns observed within dyads were different from those of cross-dyad correlations (cf. Figure 5A,B), suggesting that cross-dyad correlations of “reach-to-grasp” kinematics reflected purely average-trial coordination.

The fundamental difference between “reach-to-grasp” kinematics and timing variables was further supported on the level of cross-dyad correlations where factor analyses in four out of six experimental conditions yielded factors that loaded separately with “reach-to-grasp” kinematics and timing variables (eigenvalues > 0.5, > 80% explained variance; varimax rotation, extraction method: ordinary least squares; Supplementary Table S1). Based on this and our interest in further understanding average-trial coordination, the last step of our analysis focused on the “reach-to-grasp” kinematics.

### Dyadic Movements: Correlation Matrix Analysis of Grasp-Like Synergies

**FIGURE 7:**
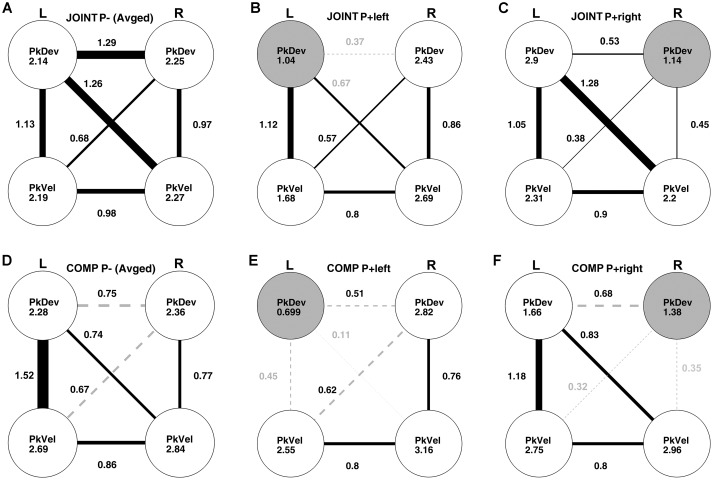
Cross-dyads correlations of kinematic variables: left hand peak deviation (L PkDev), right hand peak deviation (R PkDev), left hand peak velocity (L PkVel), right hand peak velocity (R PkVel). Correlation coefficients were Fisher’s *z*-transformed before further analyses. The correlations in conditions without perturbations (P–) were averaged. **(A)** Unperturbed JOINT P– up and down conditions. Coefficients inside disks indicate correlations between “JOINT P– up” and “JOINT P– down” conditions. Edges connecting disks graph correlations between variables for “up” and “down” on average. Line thickness and nearby coefficients reflect correlation sizes. All edges are solid black lines to indicate that they were significant as per bootstrapping analysis. **(B)** Perturbed JOINT P+ left condition. Same conventions as in panel **(A)**, except, coefficients inside disks reflect correlations between the left perturbed condition and the average unperturbed conditions; the gray colored disk indicates a significant reduction compared to the “up” vs. “down” correlation in panel **(A)**. Edges illustrate intercorrelations between variables during the perturbed left condition. Gray coefficients indicate a significant reduction relative to panel **(A)**. Gray and dashed edges indicate non-significant intercorrelations. **(C)** Perturbed JOINT P+ right condition. All conventions equivalent to panel **(B)**. **(D)** Unperturbed COMP P– up and down conditions. Same conventions as in panel **(A)**. **(E,F)** Perturbed COMP P+ left and right conditions, respectively. Same conventions as in panels **(B,C)**.

Which action features did co-actors utilize for the average-trial coordination of their “reach-to-grasp” kinematics? To answer this question, we conducted *post hoc* a cross-dyad analysis of “reach-to-grasp” kinematics with Fisher’s *z*-transformed correlations involving multiple bootstrapping steps (Supplementary Tables S2–S5 in Supplementary Material for details; also see Supplementary Figure S4 and Supplementary Tables S6, S7 for an equivalent analysis of the timing variables). Note that this analysis resembles a path analysis; unlike the latter, however, it takes into account the experimental manipulations to establish causalities. Results for the unperturbed JOINT conditions are presented in Figure 7A. The disks show the correlations of the “reach-to-grasp” kinematic variables with themselves between the two unperturbed (“up” and “down”) conditions. All these cross-condition correlations were high and significant (see Supplementary Table S2A). Also significant were all within-condition correlations between variables, graphed as edges connecting disks (correlations within “up” and “down” conditions were very similar and, thus, collapsed).

The correlational structure changed when a perturbation was introduced in the JOINT P+ left condition (Figure 7B): the perturbation selectively affected the left PkDev variable (the gray disk indicates a significantly reduced correlation with the unperturbed conditions compared to the correlation between “up” and “down”; no other correlation with the unperturbed data was reduced, Supplementary Table S2A). This effect made it possible to examine causalities between variables. The underlying logic is that if two variables A and B covary because A influences B (directly or via hidden variables), then perturbing B through a random factor should reduce the correlation whereas perturbing A should carry forward to influence B with little impact on the correlation. We found that the left person’s PkDev correlated significantly less with the right person’s PkDev (*z* = 0.37) and PkVel (*z* = 0.67) compared to the unperturbed conditions, whereas the correlation with the left PkVel stayed the same (Supplementary Tables S4A, S5A). This indicates that, without perturbation, the left person’s PkDev was influenced by their partner’s “reach-to-grasp” kinematics but not by their own velocity.

The perturbation during the JOINT P+ right condition had an equivalently selective effect on the right PkDev variable (Figure 7C). Cross-variable correlations, however, were not significantly impacted except for numerical trends (Supplementary Tables S4B, S5B). This could indicate that co-actors on the left side contributed more to the dyadic coordination whereas the right person took more of a lead. However, more research is necessary to confirm this trend.

For the COMP task (just like for JOINT), the unperturbed conditions (Figure 7D) produced significant cross-condition correlations (disks, Supplementary Table S2D) and mostly significant correlations between variables (edges, two correlations did not pass Bonferroni, see dashed edges and Supplementary Table S2D). Also, once again left and right perturbations had selective effects on the ipsilateral PkDev data (gray disks in Figure 7E,F; Supplementary Tables S3C,D), yet with different consequences: correlations of the respective PkDev with both PkVels declined, but the correlation with the other PkDev did not. To confirm that this was not due to a floor effect of modest correlations we bootstrapped the alpha errors more precisely and found them to be *p* = 0.2 and *p* = 0.39 for left and right perturbation, respectively.

## Discussion

It has been proposed that physical interactions between people as well as abstract ones, including underlying social cognition might be grounded in sensorimotor control processes that make use of internal forward models to predict one’s own muscle activity as well as another person’s actions and intentions (Wolpert et al., 2003). We reasoned that, if so, forward models should enable people to predict their partner’s movements in joint action tasks, a form of physical interpersonal interaction. To show that joint actions can be coordinated with the help of forward models, we tested a scenario where other forms of coordination could be ruled out. That is, partners would either coordinate or not at all. Specifically, we had pairs of participants perform a novel, irreducible joint lifting task that, implicitly and unbeknown to the participants, afforded movements resembling those of an individual person grasping an object and that incorporated perturbations known to specifically impact grasp movement computations (Glover et al., 2005; Tunik et al., 2005; Rice et al., 2006; Le et al., 2014; Zaal and Bongers, 2014). We found that co-actors quickly aligned their movements with emergent grasp-like trajectories. “Reach-to-grasp” kinematics and movement timing were coordinated in different ways. Timing covaried across dyads, when we looked at movements on average, as well as within each dyad from trial to trial. These observations are consistent with an important contribution of same-trial coordination. In contrast, “reach-to-grasp” kinematics only covaried across dyads with only spurious covariance from trial to trial. This suggests that dyads coordinated their “reach-to-grasp” kinematics based on offline information about one another’s movements. As we will argue, our data constitute a proof of principle that people can quickly acquire sensorimotor control for joint actions using mutual action observation to form internal representations, that is, forward models, of the dyadic “reach-to-grasp” kinematics.

We contend that the movements that people showed during the JOINT task were essentially grasp actions where each co-actor took into account the movements of his or her respective partner. There are several reasons to assume that the movements during the JOINT task truly were grasp movements. First, the JOINT task had the computational affordances of a grasp task in that it required participants to jointly lift the object. For this, sensorimotor control needs to incorporate the weight, shape and surface properties of the given object so as to compute matching grasp end points and required force (Blake and Brady, 1992).

A second reason is the ease with which participants learned the task within a few practice trials and with little change during the actual task. It suggests that participants relied on pre-existing motor programs, and it is most likely that they would have relied on relevant bimanual grasp programs than mere reach or push programs.

Third and in support of this idea, co-actors moved their hands in ways that likened grasp movements of an individual person using two hands to grasp an object – as demonstrated in our control experiment. Bimanual grasping employs similar cortical areas as grasping with one hand (Le et al., 2014, 2016) in the non-dominant right hemisphere (Le and Niemeier, 2013a,b). The finding that the current joint lifting task invokes behavior that is similar to bimanual grasping is interesting in particular because the JOINT task only required people to have their fingers positioned on opposite ends of the object and then to apply equal amounts of force. Nevertheless, the emergent behavior of the two co-actors together resembled the grasp actions of a single person (see section “Control Experiment,” also see Le and Niemeier, 2013a,b) in that they showed an MGAe during the second half of the movement, and that the MGAe was scaled to the “grasp”-relevant size of the object with a gain that approximated the proficiency of someone grasping with their non-dominant hand (Le and Niemeier, 2014). What is more, the JOINT instructions neither mentioned peak deviations nor did they require their coordination. In contrast, the COMP task did not yield grasp-like scaling of the MGAe. This does not reflect a speed-accuracy trade-off because (a) MGAe gains did not decline with velocity, (b) MGAe’s did not become more variable during the COMP task, and (c) COMP task movement were coordinated, contrary to the notion of precision being sacrificed for speed. Thus, our data indicate that co-actors only showed coordinated grasp-like movements specifically in the task that required “grasping” and lifting of the object.

As a fourth reason to assume that the JOINT movements were grasp actions, peak deviation coordination was most substantially affected when we perturbed the “grasp”-relevant dimension of the object during JOINT trials, but not during COMP trials. Such perturbations specifically affect grasp computations (Glover et al., 2005; Tunik et al., 2005; Rice et al., 2006; Le et al., 2014, 2016). In sum, we found that the JOINT task had participants behave in a synergistic manner that is best described as grasp action.

The finding of such emergent “grasp” behavior extends previous research that has shown that joint walking of two people exhibits emergent synergies much like a quadruped when the co-actors are mechanically coupled with direct somatosensory feedback (Harrison and Richardson, 2009). Our results show that synergistic behavior can arise from visual information as well. What is more, we can argue that at least for the synergies of “reach-to-grasp” kinematics co-actors must have used forward models within internal feedback systems to coordinate because all alternative explanations that do not include internal feedback can be ruled out.

First, we can rule out that direct feedback coordinated “reach-to-grasp” kinematics because, as expected, we found nearly no same-trial coordination of “reach-to-grasp” kinematics. Same-trial coordination between two partners likely is importantly governed by direct feedback where movements are controlled via online sensory information. For example, in our tasks partners could monitor one another’s timing moment by moment and coordinate accordingly. In contrast, peak velocities and peak deviations were not coordinated during the same trial, consistent with the prediction that these maxima can only be determined once the movement has been largely completed – too late for same-trial coordination. Crucially, because “reach-to-kinematics” showed little same-trial coordination, but substantial average-trial coordination, dyads must have coordinated based on motor control systems other than direct feedback alone.

Second, average-trial coordination of peak deviations reflected no cognitive strategy. It is clear that conscious cognitive control makes human actions extraordinarily flexible and even allows for actions for which no prior motor programs exist (Rossetti, 1998). However, we argue that cognitively controlled average-trial coordination of “grasping” is implausible because our participants were naive as to the purpose of the study (our instructions even avoided the term “grasping”). Therefore, it is implausible that partners would have been aware of the fact that bimanual reach-to-grasp kinematics for the left and right hand are symmetrical. In addition, there was no reason to attempt to mimic the symmetry. Also, it is doubtful that they directed much attention to their movements while monitoring the rotations of the object. Finally, it is implausible that partners consciously used “reach-to-grasp” kinematics to “communicate” with their partner about how much force they would use to push against the object during lifting. That is, there was no need to signal force information on average, given that grip force synergies can be attained through online control via somatosensory information (Solnik et al., 2016). Therefore, participants must have coordinated their grasp-like movements implicitly (that is, we argue that *how* movements curved was beyond conscious control, but not *which* movement end points were expected, see later). In contrast, during the COMP task partners coordinated reach-to-grasp kinematics arguably because they attempted to outmatch their respective partner in speed, as per instruction. Hence it is conceivable that they formed a cognitive representation of one another’s efforts that then was used to control movement velocity consciously.

Third, hybrid motor control systems can explain our data but to do so, they necessarily have to include components of internal feedback. To elaborate, the fact that perturbations disrupted coordination and increased ipsilateral peak deviations cannot be explained by a simple internal feedback system alone. Two hybrid systems are conceivable, a third is not: It is possible that, in parallel to the internal feedback system, a secondary motor program was prompted to step in whenever perturbations occurred so as to avoid collisions with the object. An anti-collision program would use direct feedback from online visual information about the object. However, on its own it would be unable to attain any dyadic average-trial coordination of peak values, as already discussed. It is also possible that people used an extended internal feedback system that integrates direct feedback. Specifically, information from sensory feedback and action observation could travel along two channels, one to train the forward model and a second channel to partake in the motor error computations.

A third hybrid system cannot explain our data: one could argue that direct feedback could respond to perturbations as well as perform coordination if sensory feedback served not only to inform motor error computations, but also as a teaching signal of the inverse model. It is indeed fair to assume that inverse models are plastic (not shown in Figure 1A). However, training inverse models is more difficult than training forward models because of more complex input/output relationships (e.g., Wolpert et al., 2003). Thus, the inverse model should be slow to learn (e.g., Latash, 1999) to compute motor commands that reflect coordination with a partner in a novel task. This is at odds with our observation that participants learned joint lifting quickly. To accelerate joint lifting training, one could further speculate that perhaps the inverse model only learned to generate motor commands that would result in more or less forceful pushing movements to match the partner’s force sensed during previous trials. Thus, the system would perform average-trial coordination of force, and any seeming coordination between peak deviations and peak velocities would be a secondary effect conveyed through some biomechanic contingency between force and peak values within each person. However, it is not clear whether such contingencies exist (e.g., Brenner and Smeets, 1996; Jackson et al., 2002; Chib et al., 2009; Danion et al., 2013). What is more, it would imply that there are fewer degrees of separation between peak data within the same person than between two people. Thus, correlations within people would always be larger than correlations between people, inconsistent with our data. In conclusion, the sole explanation that remains for average-trial-coordinated grasp-like behavior during the JOINT task is a control system that includes internal feedback and forward models.

Forward model-based coordination implies that co-actors must have formed memories of one another’s movements based on action observations (e.g., Hari et al., 1998; Flanagan and Johansson, 2003; Stefan et al., 2005; Frith and Frith, 2006) – apparently already during practice trials. The co-actors then combined these memories with efference copies for their own hand movements as inputs into a forward model to predict the state of the synergistic “grasp” (Figure 1A). It is likely that these “grasp” programs were derived from pre-existing programs that normally control bimanual grasps (e.g., Le and Niemeier, 2013a,b; Le et al., 2014), consistent with the rapid learning (e.g., Neva and Henriques, 2013).

In addition, our data provide preliminary evidence that to coordinate within the synergistic joint “grasp” system co-actors might have incorporated forward models deliberately in their planning. Accessibility to deliberate planning processes (i.e., a certain amount of cognitive penetration) is a necessary feature of forward models, should they be able to aid social or any other kind of cognitive processes (e.g., formally solving mathematical problems is probably not aided by motor control processes in the cerebellum although its processes are equivalent to solving mathematical equations). In the current study, deliberate access probably played a role in the JOINT task where co-actors curved their movements in ways that made most sense statistically. Notably, in 3 out of 4 rotation conditions one of the two co-actor’s movements would end at the same point in space (for the left co-actor after up/down/right rotations; for the right co-actor after up/down/left rotations). It seems unlikely that co-actors did not notice that rotations without ipsilateral change in end points were more common. Further, in these conditions the respective co-actor showed small peak deviations (Figure 3C) that were, across conditions, similar (Figure 7A–C). It is conceivable, therefore, that co-actors always, and deliberately planned to move their hand to the most likely end point and that they only discarded the plan if it became apparent that the rotations had altered the movement end point (left rotations for the left co-actor; right rotations for the right co-actor). In those cases co-actors still managed to increase peak deviations but coordination with their partner diminished, probably because there was no more time to switch to another coordinated movement plan. Of course, it is not impossible that co-actors remained unaware of the statistics and that their expectations formed involuntarily. To sort out the two possibilities future studies should test whether the expectations can be influenced by voluntary symbolic cues such that symbolically predicted perturbations no longer disrupt coordination.

What sources of information did co-actors use to perform average-trial coordination of grasp-like movements? Comparing unperturbed and perturbed JOINT trials revealed some of the causalities within the correlational structure of the tasks. Given the disruptions in correlations we conclude that at least co-actors on the left must have chosen their “grasp” actions, as reflected in the peak deviations, by memorizing their partner’s movement curvatures and velocity profiles (e.g., Kilner et al., 2003; Stefan et al., 2005).

During competition, however, causalities changed. Peak deviations no longer influenced one another but depended on the respective person’s own peak velocities. This shows that participants used different motor programs where velocity coordination (probably reflecting matched competitive efforts) coordinated peak deviations indirectly.

Unlike average-trial coordination, same-trial coordination of “reach-to-grasp” kinematics was spurious and, thus, too small to explain average-trial coordination (in contrast to the sizeable same-trial coordination of the timing variables). Nevertheless, “reach-to-grasp” kinematics did show significant same-trial coordination. Most notably, same-trial coordination of peak deviations yielded negative correlations, that is, trial by trial participants tended to move their arms left and right as their partner moved in the same direction. This form of coordination is at odds with average-trial coordination where movements coordinated in mirror-symmetric directions, suggesting that action observations of reach movements “spilled” into people’s own action control involuntarily (e.g., Schmidt et al., 1990; Watkins et al., 2003; Oullier et al., 2008; Fine and Amazeen, 2011) and contrary to the overarching goal of “grasp” coordination.

## Conclusion

In conclusion, using a novel joint lifting task we found that co-actors showed emergent joint “grasp” behavior. The movements relied on action observations to attain average-trial (likely deliberate) forms of coordination as an indicator of internal feedback. The study provides important proof for the long-held assumption that human sensorimotor control uses forward models to compute interaction with other people. As such the sensorimotor system appears to be one of the sources that have given rise to social cognition.

## Ethics Statement

This study was carried out in accordance with the recommendations of the Human Participants Review Sub-Committee of the University of Toronto with written informed consent from all subjects. All subjects gave written informed consent in accordance with the Declaration of Helsinki. The protocol was approved by the Human Participants Review Sub-Committee of the University of Toronto.

## Author Contributions

LG and MN conceived and designed the work, collected, analyzed and interpreted the data, drafted and critically revised the article, and approved the final version to be published. NP collected, analyzed, and interpreted the data.

## Conflict of Interest Statement

The authors declare that the research was conducted in the absence of any commercial or financial relationships that could be construed as a potential conflict of interest.
