# Supplementary material for: Emergent Synergistic Grasp-Like Behavior in a Visuomotor Joint Action Task: Evidence for Internal Forward Models as Building Blocks of Human Interactions
Source: Front Hum Neurosci. 2019 Feb 6;13:37. doi: 10.3389/fnhum.2019.00037 (PMC6372946; doi:10.3389/fnhum.2019.00037)
Supplement: Supplementary file 1 [file Data_Sheet_1.docx]

**Supplementary Figure Captions and Tables**

***Figure S1.*** (A) Peak deviations of the left (LH) and right (RH) hands during bimanual grasping. (B) Peak velocities of the left and right hands during bimanual grasping. (C) Peak deviation times of the left and right hands during bimanual grasping. (D) Peak velocity times of the left and right hands during bimanual grasping.

***Figure S2.*** Cross-subject correlations of (A) peak deviation (PkDev), (B) peak velocity (PkVel), (C) time of peak deviation (tPkDev), and (D) time of peak velocity (tPkVel) within time windows gliding across trials. Black lines: no perturbation (P-) conditions. Grey lines: perturbed (P+) conditions. Error wraps indicate 95% confidence intervals.

***Figure S3.*** Cross-dyad correlations of (A) peak deviation (PkDev), (B) peak velocity (PkVel), (C) time of peak deviation (tPkDev), and (D) time of peak velocity (tPkVel) within time windows gliding across trials. Black lines: joint lifting (JOINT) conditions. Grey lines: competition (COMP) condition. Solid lines: no perturbation (P-) conditions. Dash lines: perturbed (P+) conditions. Error wraps indicate 95% confidence intervals.

***Figure S4.*** Cross-dyad correlations of left hand time of peak deviation (L tPkDev), right hand time of peak deviation (R tPkDev), left hand time of peak velocity (L tPkVel), right hand time of peak velocity (R tPkVel) in all conditions. Correlation coefficients were Fisher’s z-transformed before further analyses. The correlations in conditions without perturbations (P-) were averaged. (A) Unperturbed JOINT P- up and down conditions. Coefficients inside disks indicate correlations between “JOINT P- up” and “JOINT P- down” conditions. Edges connecting disks graph correlations between variables for “up” and “down” on average. Line thickness and nearby coefficients reflect correlation sizes. All edges are solid black lines to indicate that they were significant as per bootstrapping analysis. (B) Perturbed JOINT P+ left condition. Same conventions as in A, except, coefficients inside disks reflect correlations between the left perturbed condition and the average unperturbed conditions; the grey coloured disk indicates a significant reduction compared to the “up” vs. “down” correlation in A. Edges illustrate intercorrelations between variables during the perturbed left condition. Grey coefficients indicate a significant reduction relative to A. Grey and dashed edges indicate non-significant intercorrelations. (C) Perturbed JOINT P+ right condition. All conventions equivalent to B. (D) Unperturbed COMP P- up and down conditions. Same conventions as in A. (E) & (F) Perturbed COMP P+ left and right conditions, respectively. Same conventions as in B&C.

| **Table S1**  *Factor Analyses of Kinematics and Timing Variables of the Left and Right Hands in Each Condition*  *Minimum Eigenvalue: 0.5* | | | | | | | | | |
| --- | --- | --- | --- | --- | --- | --- | --- | --- | --- |
|  | | Rotated Factor Loadings | | | | | | | |
| Factors | Cumulative % Variance Explained | Left tPkDev | Right tPkDev | Left tPkVel | Right tPkVel | Left PkDev | Right PkDev | Left PkVel | Right PkVel |
| Joint P- | | | | | | | | | |
| 1 | 82.210 | .931 | .924 | .943 | .898 | -.904 | -.820 | -.830 | -.885 |
| Joint P+ Left | | | | | | | | | |
| 1 | 73.759 | .717 | .880 | .831 | .922 |  | -.682 |  | -.704 |
| 2 | 85.804 | -.641 | -.343 | -.460 |  | .715 | .307 | .968 | .489 |
| Joint P+ Right | | | | | | | | | |
| 1 | 77.793 | .909 | .955 | .947 | .911 | -.813 |  | -.891 | -.833 |
| 2 | 87.916 |  |  |  |  | .361 | .970 |  | .305 |
| COMP P- | | | | | | | | | |
| 1 | 64.109 | -.357 |  |  |  | .896 | .704 | .838 | .661 |
| 2 | 83.182 | .421 | .913 | .459 | .850 |  |  |  | -.641 |
| 3 | 91.488 | .825 | .374 | .839 | .334 | -.413 |  |  |  |
| COMP P+ Left | | | | | | | | | |
| 1 | 61.378 | .405 | .895 | .422 | .773 |  |  | -.418 | -.799 |
| 2 | 80.429 | .876 | .466 | .884 | .473 |  |  | -.411 |  |
| 3 | 88.677 |  |  |  |  | .615 | .796 | .579 | .399 |
| COMP P+ Right | | | | | | | | | |
| 1 | 65.997 | .424 | .777 | .351 | .873 | -.442 |  | -.527 | -.527 |
| 2 | 82.153 | .785 | .470 | .896 | .453 | -.324 |  |  |  |
| 3 | 90.566 | -.376 | -.346 |  |  | .796 | .693 | .579 | .414 |

| **Table S2**  *Correlations Within Conditions (Edges) and Across Conditions (Discs)* | | | | | | | |
| --- | --- | --- | --- | --- | --- | --- | --- |
| Section A: JOINT P- | | | | | | | |
| Edges | | | | | | | |
| Serial Bonf | Lower bound | Left | Top | Diag 1 | Diag 2 | Bottom | Right |
| 1 | 2.5% / 6 | **0.442** | **0.827** | **0.664** | **0.080** | **0.241** | **0.533** |
|  |  | ***** | ***** | ***** | ***** | ***** | ***** |
| Discs | | | | | | | |
| Serial Bonf | Lower bound | L.PkDev | R.PkDev | L.PkVel | R.PkVel |  |  |
| 1 | 2.5% / 4 | **1.634** | **1.425** | **1.672** | **1.361** |  |  |
|  |  | * | * | * | * |  |  |
| Section B: JOINT P+ (Left) | | | | | | | |
| Edges | | | | | | | |
| Serial Bonf | Lower bound | Left | Top | Diag 1 | Diag 2 | Bottom | Right |
| 1 | 2.5% / 6 | **0.443** | -0.341 | -0.167 | -0.003 | -0.009 | **0.444** |
| 2 | 2.5% / 5 | **0.460** | -0.301 | -0.148 | **0.026** | **0.008** |  |
| 3 | 2.5% / 4 |  | -0.275 | -0.115 | **0.053** | **0.035** |  |
| 4 | 2.5% / 3 |  | -0.242 | -0.055 |  | **0.068** |  |
| 5 | 2.5% / 2 |  | -0.192 | **0.012** |  |  |  |
| 6 | 2.5% |  | -0.097 |  |  |  |  |
|  |  | * | **n.s.** | * | * | * | * |
| Discs | | | | | | | |
| Serial Bonf | Lower bound | L.PkDev | R.PkDev | L.PkVel | R.PkVel |  |  |
| 1 | 2.5% / 4 | **0.231** | **1.960** | **1.109** | **1.673** |  |  |
|  |  | * | * | * | * |  |  |
| Section C: JOINT P+ (Right) | | | | | | | |
| Edges | | | | | | | |
| Serial Bonf | Lower bound | Left | Top | Diag 1 | Diag 2 | Bottom | Right |
| 1 | 2.5% / 6 | **0.417** | **0.163** | **0.236** | -0.001 | **0.280** | **0.089** |
| 2 | 2.5% / 5 |  | **0.190** | **0.294** | **0.009** | **0.298** | **0.098** |
|  |  | * | * | * | * | * | * |
| Discs | | | | | | | |
| Serial Bonf | Lower bound | L.PkDev | R.PkDev | L.PkVel | R.PkVel |  |  |
| 1 | 2.5% / 6 | **2.128** | **0.361** | **1.585** | **1.103** |  |  |
|  |  | * | * | * | * |  |  |
| Section D: COMP P- | | | | | | | |
| Edges | | | | | | | |
| Serial Bonf | Lower bound | Left | Top | Diag 1 | Diag 2 | Bottom | Right |
| 1 | 2.5% / 6 | **0.570** | -0.217 | **0.235** | -0.269 | **0.414** | **0.065** |
| 2 | 2.5% / 5 |  | -0.192 | **0.253** | -0.250 | **0.428** | **0.086** |
| 3 | 2.5% / 4 |  | -0.158 | **0.272** | -0.203 |  | **0.110** |
| 4 | 2.5% / 3 |  | -0.129 |  | -0.150 |  | **0.148** |
| 5 | 2.5% / 2 |  | -0.073 |  | -0.087 |  |  |
|  |  | * | **n.s.** | * | **n.s.** | * | * |
| Discs | | | | | | | |
| Serial Bonf | Lower bound | L.PkDev | R.PkDev | L.PkVel | R.PkVel |  |  |
| 1 | 2.5% / 4 | **1.256** | **1.727** | **1.780** | **2.044** |  |  |
|  |  | * | * | * | * |  |  |
| Section E: COMP P+ (Left) | | | | | | | |
| Edges | | | | | | | |
| Serial Bonf | Lower bound | Left | Top | Diag 1 | Diag 2 | Bottom | Right |
| 1 | 2.5% / 6 | -0.622 | -0.777 | -0.668 | -0.311 | **0.264** | **0.158** |
| 2 | 2.5% / 5 | -0.578 | -0.686 | -0.646 | -0.292 |  | **0.178** |
| 3 | 2.5% / 4 | -0.542 | -0.605 | -0.608 | -0.261 |  |  |
|  |  | n.s. | n.s. | n.s. | n.s. | * | * |
| Discs | | | | | | | |
| Serial Bonf | Lower bound | L.PkDev | R.PkDev | L.PkVel | R.PkVel |  |  |
| 1 | 2.5% / 4 | -0.192 | **1.982** | **1.502** | **2.483** |  |  |
| 2 | 2.5% / 3 | -0.133 | **2.059** | **1.590** |  |  |  |
| 3 | 2.5% / 2 | -0.071 |  | **1.658** |  |  |  |
| 4 | 2.5% | **0.067** |  |  |  |  |  |
|  |  | * | * | * | * |  |  |
| Section F: COMP P+ (Right) | | | | | | | |
| Edges | | | | | | | |
| Serial Bonf | Lower bound | Left | Top | Diag 1 | Diag 2 | Bottom | Right |
| 1 | 2.5% / 6 | **0.382** | -0.282 | **0.289** | -0.619 | **0.312** | -0.660 |
| 2 | 2.5% / 5 | **0.418** | -0.241 | **0.300** | -0.595 | **0.330** | -0.601 |
| 3 | 2.5% / 4 | **0.457** | -0.208 | **0.339** | -0.514 | **0.362** | -0.540 |
| 4 | 2.5% / 3 | **0.516** | -0.167 | **0.368** | -0.449 | **0.398** | -0.302 |
|  |  | * | **n.s.** | * | **n.s.** | * | **n.s.** |
| Discs | | | | | | | |
| Serial Bonf | Lower bound | L.PkDev | R.PkDev | L.PkVel | R.PkVel |  |  |
| 1 | 2.5% / 4 | **1.025** | **0.339** | **2.168** | **2.048** |  |  |
|  |  | * | * | * | * |  |  |

*Note*. Step 1 of the analysis examined the cross-dyads correlational structure of the PkVel and PkDev variables in all conditions (see Figure 6). Displayed values are z-transformed lower bounds of the bootstrapped confidence intervals (95%; two-tailed) and are corrected using the Multistage Bonferroni procedure. All upper bounds are above 0, thus lower bounds above 0 are considered as significant. Stage 1 of the Bonferroni procedure tested the lower bounds obtained at the strictest percentile rank (2.5 / the number of variables). If the largest lower bound is significant, the next stage tests the lower bounds obtained at a less strict percentile rank by reducing the number of variables by 1, until the first nonsignificant lower bound. (A) unperturbed JOINT task. “Disc correlations” were calculated between the unperturbed “up” and “down” conditions. “Edge correlations” were collapsed across unperturbed conditions. (B) left perturbed JOINT task. Discs show correlations with the unperturbed condition for the respective variable. (C) right perturbed JOINT task. Same conventions as (B). (D)-(F) COMP task. Same conventions as (A)-(C), respectively. “Edges” and “discs” indicate within and cross condition correlations, respectively. Left: “left edge correlation” between left PkDev and left PkVel. Top: “top edge correlation” between left PkDev and right PkDev. Diag 1: “diagonal 1 edge correlation” between left PkDev and right PkVel. Diag 2: “diagonal 2 edge correlation” between Left PkVel and Right PkDev. Bottom: “bottom edge correlation” between left PkVel and right PkVel. Right: “right edge correlation” between right PkDev and right PkVel. Significant lower-bounds are bolded and highlighted in grey. The asterisks at the bottom of each table cluster indicate significance.

| **Table S3**  *Disc Reduction from Standard Conditions (JOINT P- & COMP P-)* | | | | | |
| --- | --- | --- | --- | --- | --- |
| Section A: JOINT P+ (Left) | | | | | |
| Serial Bonf | Lower bound | L.PkDev | R.PkDev | L.PkVel | R.PkVel |
| 1 | 5% / 4 | **0.165** | -1.161 | -0.386 | -1.294 |
| 2 | 5% / 3 |  | -1.116 | -0.324 | -1.208 |
|  |  | * | n.s. | n.s. | n.s. |
| Section B: JOINT P+ (Right) | | | | | |
| Serial Bonf | Lower bound | L.PkDev | R.PkDev | L.PkVel | R.PkVel |
| 1 | 5% / 4 | -1.643 | **0.529** | -0.955 | -0.547 |
| 2 | 5% / 3 | -1.587 |  | -0.877 | -0.503 |
|  |  | n.s. | * | n.s. | n.s. |
| Section C: COMP P+ (Left) | | | | | |
| Serial Bonf | Lower bound | L.PkDev | R.PkDev | L.PkVel | R.PkVel |
| 1 | 5% / 4 | **0.132** | -1.717 | -0.558 | -1.382 |
| 2 | 5% / 3 |  | -1.628 | -0.513 | -1.285 |
|  |  | * | n.s. | n.s. | n.s. |
| Section D: COMP P+ (Right) | | | | | |
| Serial Bonf | Lower bound | L.PkDev | R.PkDev | L.PkVel | R.PkVel |
| 1 | 5% / 4 | -0.295 | **0.409** | -1.575 | -1.009 |
| 2 | 5% / 3 | -0.205 |  | -1.469 | -0.966 |
|  |  | n.s. | * | n.s. | n.s. |

*Note*. Step 2 of the analysis of the cross-dyads correlational structure tested the expected impact of perturbations on discs, i.e., whether perturbations reduced cross-condition correlations relative to the standard P- conditions. (A) Impact of left perturbations on the cross-condition correlations of PkDev and PkVel (Discs) relative to the unperturbed JOINT condition. (B) Impact of right perturbations on the Discs relative to JOINT P-. (C) Impact of left perturbations on the Discs of PkDev relative to the unperturbed COMP condition. (D) Impact of right perturbations on the discs relative to COMP P-. A multistage Bonferroni corrected bootstrap analysis was performed equivalent to Table S2.

| **Table S4**  *Averaged Edge Reduction from Standard Conditions (JOINT P- & COMP P-)* | | | | | |
| --- | --- | --- | --- | --- | --- |
| Section A: JOINT P+ (Left) | | | | | |
| Serial Bonf | Lower bound | L.PkDev | R.PkDev | L.PkVel | R.PkVel |
| 1 | 5% | **0.245** | N/A | N/A | N/A |
|  |  | * |  |  |  |
| Section B: JOINT P+ (Right) | | | | | |
| Serial Bonf | Lower bound | L.PkDev | R.PkDev | L.PkVel | R.PkVel |
| 1 | 5% | N/A | **0.071** | N/A | N/A |
|  |  |  | * |  |  |
| Section C: COMP P+ (Left) | | | | | |
| Serial Bonf | Lower bound | L.PkDev | R.PkDev | L.PkVel | R.PkVel |
| 1 | 5% | **0.354** | N/A | N/A | N/A |
|  |  | * |  |  |  |
| Section D: COMP P+ (Right) | | | | | |
| Serial Bonf | Lower bound | L.PkDev | R.PkDev | L.PkVel | R.PkVel |
| 1 | 5% | N/A | **0.052** | N/A | N/A |
|  |  |  | * |  |  |

*Note*. Step 3 of the analysis on the cross-dyads correlational structure. Impact of perturbations on averaged within-condition correlations (edges) connecting to a given disc. Average correlations were only tested if a disc was significantly impacted by perturbations (Table S3). (A) Impact of left perturbations on the averaged edges of left PkDev relative to the unperturbed JOINT condition. (B) Impact of right perturbations on the averaged edges of right PkDev relative to JOINT P-. (C) Impact of left perturbations on the averaged edges of left PkDev relative to the unperturbed COMP condition. (D) Impact of right perturbations on the averaged edges of right PkDev relative to COMP P-. A multistage Bonferroni corrected bootstrap analysis was performed equivalent to Table S2.

| **Table S5**  *Edge Reduction from Standard Conditions (JOINT P- & COMP P-)* | | | | | | | |
| --- | --- | --- | --- | --- | --- | --- | --- |
| Section A: JOINT P+ (Left) | | | | | | | |
| Serial Bonf | Lower bound | Left | Top | Diag 1 | Diag 2 | Bottom | Right |
| 1 | 5% / 3 | -0.545 | **0.337** | **0.162** | N/A | N/A | N/A |
| 2 | 5% / 2 | -0.497 |  | **0.212** |  |  |  |
| 3 | 5% | -0.414 |  |  |  |  |  |
|  |  | n.s. | * | * |  |  |  |
| Section B: JOINT P+ (Right) | | | | | | | |
| Serial Bonf | Lower bound | Left | Top | Diag 1 | Diag 2 | Bottom | Right |
| 1 | 5% / 3 | N/A | -0.082 | N/A | -0.380 | N/A | -0.298 |
|  |  |  | **n.s.** |  | **n.s.** |  | **n.s.** |
| Section C: COMP P+ (Left) | | | | | | | |
| Serial Bonf | Lower bound | Left | Top | Diag 1 | Diag 2 | Bottom | Right |
| 1 | 5% / 3 | **0.419** | -0.582 | **0.310** | N/A | N/A | N/A |
| 2 | 5% / 2 |  | -0.511 | **0.347** |  |  |  |
| 3 | 5% |  | -0.371 |  |  |  |  |
|  |  | * | n.s. | * |  |  |  |
| Section D: COMP P+ (Right) | | | | | | | |
| Serial Bonf | Lower bound | Left | Top | Diag 1 | Diag 2 | Bottom | Right |
| 1 | 5% / 3 | N/A | -0.468 | N/A | **0.016** | N/A | **0.106** |
| 2 | 5% / 2 |  | -0.425 |  | **0.043** |  |  |
| 3 | 5% |  | -0.342 |  |  |  |  |
|  |  |  | **n.s.** |  | * |  | * |

*Note*. Step 4 of the analysis on the cross-dyads correlational structure. For any averaged edges that were found to be significant in step 3 (Table S4), the contribution of the individual edges was tested. (A) Impact of left perturbations on the edges of left PkDev relative to the unperturbed JOINT Condition. (B) Impact of right perturbations on the edges of right PkDev relative to Joint P-. (C) Impact of left perturbations on the edges of left PkDev relative to the unperturbed COMP condition. (D) Impact of right perturbations on the edges of Right PkDev relative to COMP P-. A multistage Bonferroni corrected bootstrap analysis was performed equivalent to Table S2.

| **Table S6**  *Correlations Within Conditions (Edges) and Across Conditions (Discs)* | | | | | | | |
| --- | --- | --- | --- | --- | --- | --- | --- |
| Section A: JOINT P- | | | | | | | |
| Edges | | | | | | | |
| Serial Bonf | Lower bound | Left | Top | Diag 1 | Diag 2 | Bottom | Right |
| 1 | 2.5% / 6 | **1.544** | **0.610** | **0.575** | **0.679** | **0.777** | **1.085** |
|  |  | ***** | ***** | ***** | ***** | ***** | ***** |
| Discs | | | | | | | |
| Serial Bonf | Lower bound | L.tPkDev | R.tPkDev | L.tPkVel | R.tPkVel |  |  |
| 1 | 2.5% / 4 | **1.213** | **0.942** | **1.581** | **0.872** |  |  |
|  |  | * | * | * | * |  |  |
| Section B: JOINT P+ (Left) | | | | | | | |
| Edges | | | | | | | |
| Serial Bonf | Lower bound | Left | Top | Diag 1 | Diag 2 | Bottom | Right |
| 1 | 2.5% / 6 | **1.221** | **0.404** | **0.422** | **0.542** | **0.764** | **1.147** |
|  |  | * | * | * | * | * | * |
| Discs | | | | | | | |
| Serial Bonf | Lower bound | L.tPkDev | R.tPkDev | L.tPkVel | R.tPkVel |  |  |
| 1 | 2.5% / 4 | **1.437** | **1.221** | **1.852** | **1.139** |  |  |
|  |  | * | * | * | * |  |  |
| Section C: JOINT P+ (Right) | | | | | | | |
| Edges | | | | | | | |
| Serial Bonf | Lower bound | Left | Top | Diag 1 | Diag 2 | Bottom | Right |
| 1 | 2.5% / 6 | **1.206** | **1.208** | **0.535** | **1.102** | **1.111** | **0.957** |
|  |  | * | * | * | * | * | * |
| Discs | | | | | | | |
| Serial Bonf | Lower bound | L.tPkDev | R.tPkDev | L.tPkVel | R.tPkVel |  |  |
| 1 | 2.5% / 6 | **1.620** | **0.804** | **1.712** | **1.579** |  |  |
|  |  | * | * | * | * |  |  |
| Section D: COMP P- | | | | | | | |
| Edges | | | | | | | |
| Serial Bonf | Lower bound | Left | Top | Diag 1 | Diag 2 | Bottom | Right |
| 1 | 2.5% / 6 | **1.615** | **0.392** | -0.017 | **0.370** | -0.013 | **0.775** |
| 2 | 2.5% / 5 |  | **0.418** | **-0.000** | **0.383** | **0.006** | **0.867** |
| 3 | 2.5% / 4 |  | **0.438** | **0.020** | **0.412** | **0.032** |  |
|  |  | * | ***** | * | ***** | * | * |
| Discs | | | | | | | |
| Serial Bonf | Lower bound | L.tPkDev | R.tPkDev | L.tPkVel | R.tPkVel |  |  |
| 1 | 2.5% / 4 | **1.315** | **0.769** | **1.444** | **0.972** |  |  |
|  |  | * | * | * | * |  |  |
| Section E: COMP P+ (Left) | | | | | | | |
| Edges | | | | | | | |
| Serial Bonf | Lower bound | Left | Top | Diag 1 | Diag 2 | Bottom | Right |
| 1 | 2.5% / 6 | **1.449** | **0.193** | -0.02 | **0.152** | -0.057 | **1.049** |
| 2 | 2.5% / 5 |  | **0.238** | **0.017** | **0.174** | -0.032 | **1.077** |
| 3 | 2.5% / 4 |  | **0.290** | **0.079** | **0.208** | **0.002** |  |
|  |  | * | * | * | * | * | * |
| Discs | | | | | | | |
| Serial Bonf | Lower bound | L.tPkDev | R.tPkDev | L.tPkVel | R.tPkVel |  |  |
| 1 | 2.5% / 4 | **1.255** | **0.595** | **1.570** | **0.655** |  |  |
|  |  | * | * | * | * |  |  |
| Section F: COMP P+ (Right) | | | | | | | |
| Edges | | | | | | | |
| Serial Bonf | Lower bound | Left | Top | Diag 1 | Diag 2 | Bottom | Right |
| 1 | 2.5% / 6 | **1.396** | **0.528** | **0.133** | **0.411** | -0.026 | **0.254** |
| 2 | 2.5% / 5 |  | **0.547** | **0.161** | **0.439** | **0.010** | **0.270** |
|  |  | * | * | * | * | * | ***** |
| Discs | | | | | | | |
| Serial Bonf | Lower bound | L.tPkDev | R.tPkDev | L.tPkVel | R.tPkVel |  |  |
| 1 | 2.5% / 4 | **1.731** | **0.788** | **1.856** | **0.666** |  |  |
|  |  | * | * | * | * |  |  |

*Note*. Step 1 of the analysis on the cross-dyad correlational structure of the timing variables tPkVel and tPkDev (see Supplementary Figure 4). Correlations are z-transformed lower bounds of the bootstrapped confidence intervals (95%; two-tailed) and are corrected using the Multistage Bonferroni procedure. All upper bounds are above 0, thus lower bounds above 0 are considered as significant. Stage 1 of the Bonferroni procedure tested the lower bounds obtained at the strictest percentile rank (2.5 / the number of variables). If the largest lower bound is significant, the next stage tests the lower bounds obtained at a less strict percentile rank by reducing the number of variables by 1, until the first nonsignificant lower bound. (A) unperturbed JOINT task. “Disc correlations” were calculated between the unperturbed “up” and “down” conditions. “Edge correlations” were collapsed across unperturbed conditions. (B) left perturbed JOINT task. Discs show correlations with the unperturbed condition for the respective variable. (C) right perturbed JOINT task. Same conventions as (B). (D)-(F) COMP task. Same conventions as (A)-(C), respectively. “Edges” and “discs” indicate within and cross condition correlations, respectively. Left: “left edge correlation” between left tPkDev and left tPkVel. Top: “top edge correlation” between left tPkDev and right tPkDev. Diag 1: “diagonal 1 edge correlation” between left tPkDev and right tPkVel. Diag 2: “diagonal 2 edge correlation” between Left tPkVel and Right tPkDev. Bottom: “bottom edge correlation” between left tPkVel and right tPkVel. Right: “right edge correlation” between right tPkDev and right tPkVel. Significant lower-bounds are bolded and colored in grey. The asterisks at the bottom of each table cluster indicate significance.

| **Table S7**  *Disc Reduction from Standard Conditions (JOINT P- & COMP P-)* | | | | | |
| --- | --- | --- | --- | --- | --- |
| Section A: JOINT P+ (Left) | | | | | |
| Serial Bonf | Lower bound | L.tPkDev | R.tPkDev | L.tPkVel | R.tPkVel |
| 1 | 5% / 4 | -0.890 | -1.240 | -1.451 | -1.063 |
|  |  | n.s. | n.s. | n.s. | n.s. |
| Section B: JOINT P+ (Right) | | | | | |
| Serial Bonf | Lower bound | L.tPkDev | R.tPkDev | L.tPkVel | R.tPkVel |
| 1 | 5% / 4 | -1.144 | -0.857 | -1.215 | -1.690 |
|  |  | n.s. | n.s. | n.s. | n.s. |
| Section C: COMP P+ (Left) | | | | | |
| Serial Bonf | Lower bound | L.tPkDev | R.tPkDev | L.tPkVel | R.tPkVel |
| 1 | 5% / 4 | -1.204 | -0.880 | -1.036 | -1.179 |
|  |  | n.s. | n.s. | n.s. | n.s. |
| Section D: COMP P+ (Right) | | | | | |
| Serial Bonf | Lower bound | L.tPkDev | R.tPkDev | L.tPkVel | R.tPkVel |
| 1 | 5% / 4 | -1.046 | -1.459 | -1.235 | -1.000 |
|  |  | n.s. | n.s. | n.s. | n.s. |

*Note*. Step 2 of the analysis on the cross-dyad correlational structure of the timing variables. Cross-condition impact of perturbations on discs. (A) Impact of left perturbations on the cross-condition correlations of PkDev and PkVel (Discs) relative to the unperturbed JOINT condition. (B) Impact of right perturbations on the Discs relative to JOINT P-. (C) Impact of left perturbations on the Discs of PkDev relative to the unperturbed COMP condition. (D) Impact of right perturbations on the discs relative to COMP P-. A multistage Bonferroni corrected bootstrap analysis was performed equivalent to Table S6. However, here the upper bounds for L.tPkDev and L.tPkVel in COMP P+ (Right) are below 0 (-0.043 and -0.114 respectively), and thus lower bounds below 0 for these two variables are considered as significant.
