# Supplementary figures and images for: Emergent Synergistic Grasp-Like Behavior in a Visuomotor Joint Action Task: Evidence for Internal Forward Models as Building Blocks of Human Interactions
Source: Front Hum Neurosci. 2019 Feb 6;13:37. doi: 10.3389/fnhum.2019.00037 (PMC6372946; doi:10.3389/fnhum.2019.00037)

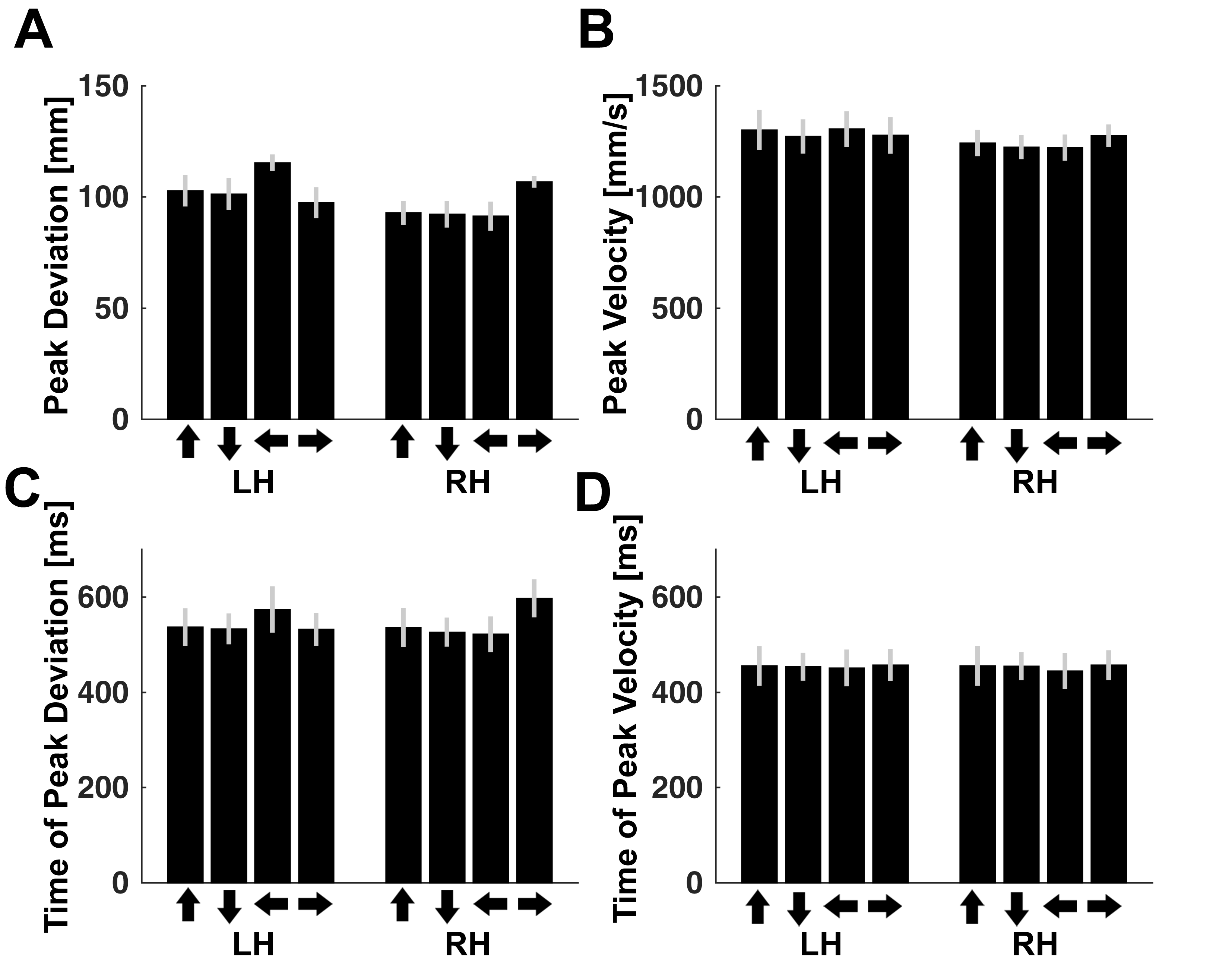

Supplement: Supplementary file 2 [file Image_1.TIF]

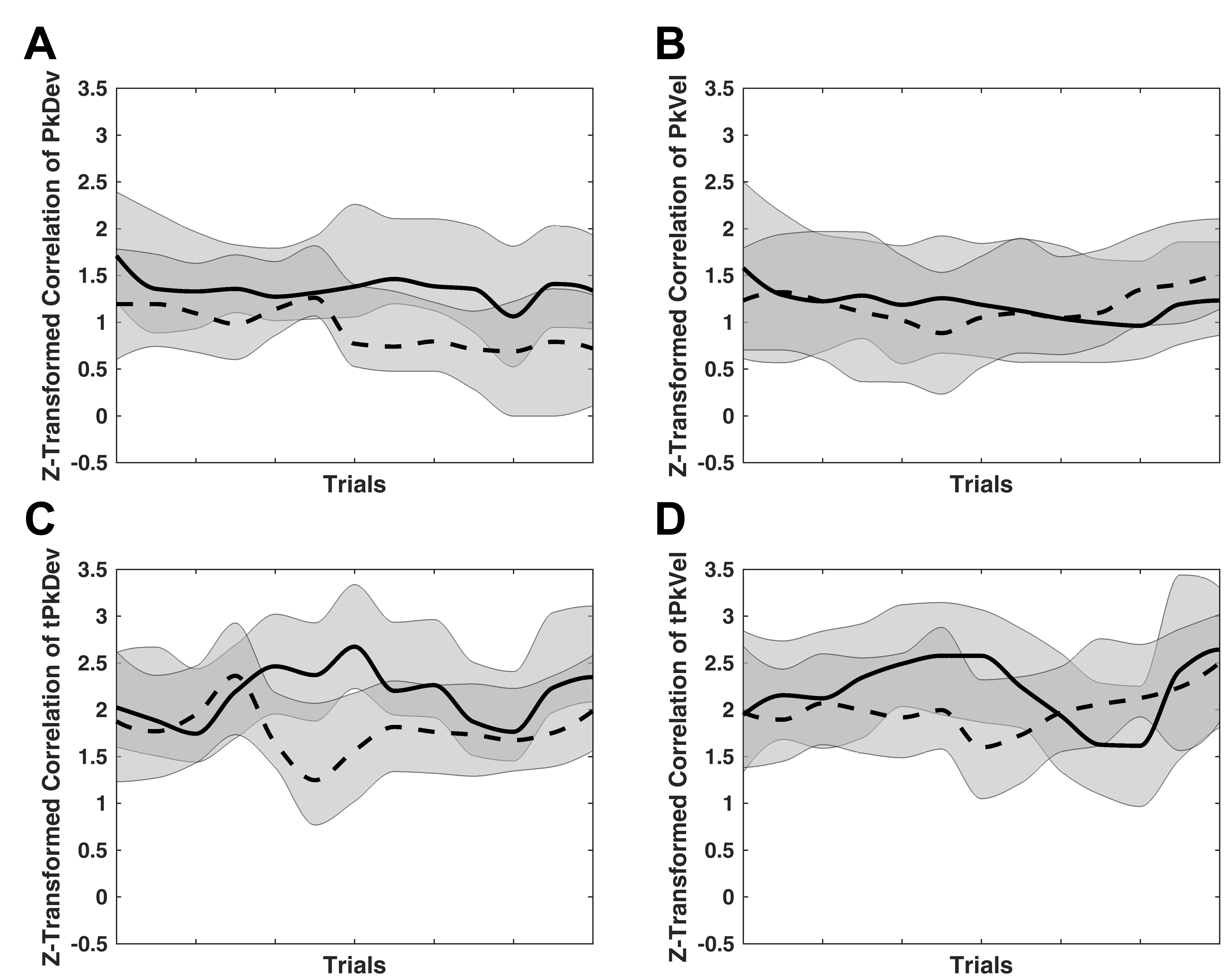

Supplement: Supplementary file 3 [file Image_2.TIF]

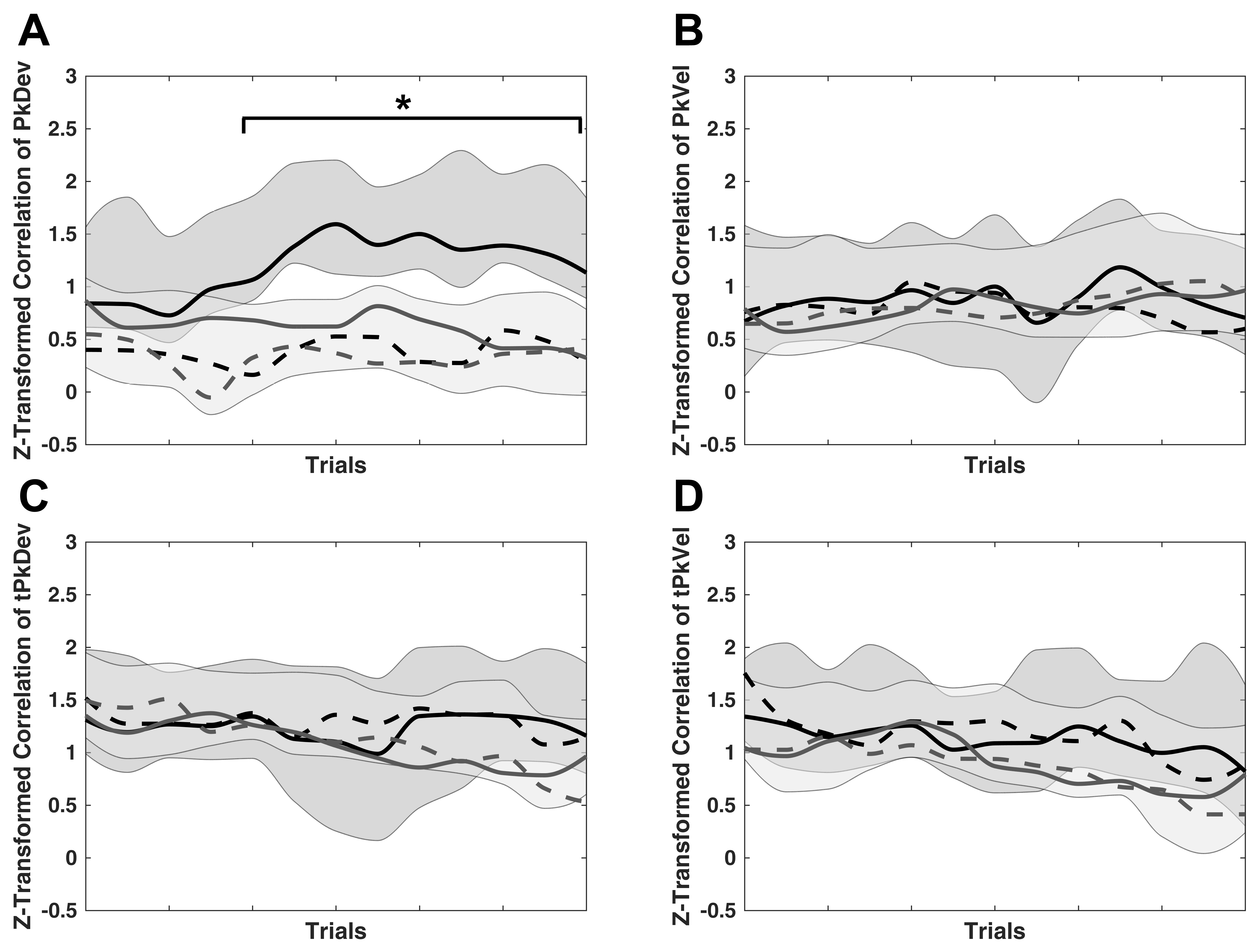

Supplement: Supplementary file 4 [file Image_3.TIF]

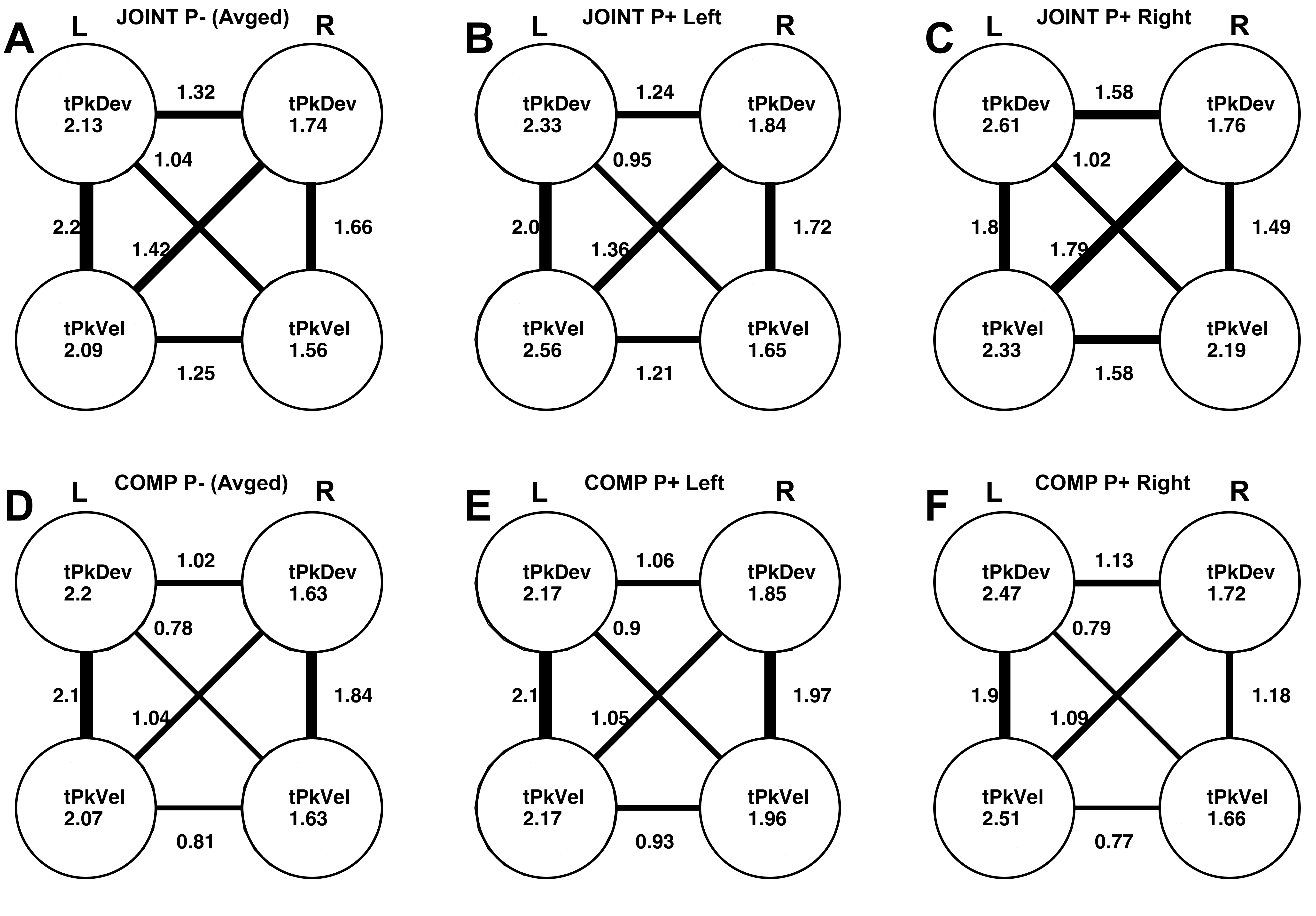

Supplement: Supplementary file 5 [file Image_4.TIF]
